# Movement syndromes of a Neotropical frugivorous bat inhabiting heterogeneous landscapes in Brazil

**DOI:** 10.1186/s40462-021-00266-6

**Published:** 2021-07-07

**Authors:** Patricia Kerches-Rogeri, Danielle Leal Ramos, Jukka Siren, Beatriz de Oliveira Teles, Rafael Souza Cruz Alves, Camila Fátima Priante, Milton Cezar Ribeiro, Márcio Silva Araújo, Otso Ovaskainen

**Affiliations:** 1 Departamento de Biodiversidade, Universidade Estadual Paulista – UNESP, Avenida 24 A,1515, Rio Claro, São Paulo, Brazil; 2 Faculty of Biological and Environmental Sciences, University of Helsinki, P.O. Box 65, Viikinkaari 1, 00014 Helsinki, Finland; 3 Department of Computer Science, Aalto University, Espoo, Finland; 4 Centre for Biodiversity Dynamics, Department of Biology, Norwegian University of Science and Technology, N-7491 Trondheim, Norway

**Keywords:** Movement behavior, Diffusion model, Individual specialization, Habitat fragmentation, Frugivory, Phyllostomidae, Seed dispersal, Space use

## Abstract

**Background:**

There is growing evidence that individuals within populations can vary in both habitat use and movement behavior, but it is still not clear how these two relate to each other. The aim of this study was to test if and how individual bats in a *Stunira lilium* population differ in their movement activity and preferences for landscape features in a correlated manner.

**Methods:**

We collected data on movements of 27 individuals using radio telemetry. We fitted a heterogeneous-space diffusion model to the movement data in order to evaluate signals of movement variation among individuals.

**Results:**

*S. lilium* individuals generally preferred open habitat with *Solanum* fruits, regularly switched between forest and open areas, and showed high site fidelity. Movement variation among individuals could be summarized in four movement syndromes: (1) average individuals, (2) forest specialists, (3) explorers which prefer *Piper*, and (4) open area specialists which prefer *Solanum* and *Cecropia*.

**Conclusions:**

Individual preferences for landscape features plus food resource and movement activity were correlated, resulting in different movement syndromes. Individual variation in preferences for landscape elements and food resources highlight the importance of incorporating explicitly the interaction between landscape structure and individual heterogeneity in descriptions of animal movement.

**Supplementary Information:**

The online version contains supplementary material available at 10.1186/s40462-021-00266-6.

## Background

Movement is defined as the change in the spatial location of an organism over time, and it has a crucial role in determining the fates of individuals, and consequently the structure and dynamics of populations, communities and ecosystems [1]. Habitat fragmentation and climate change have pervasive impacts on ecosystem maintenance [2]. These anthropogenic processes can influence directly the movement behavior of individuals and species. For example, a survey of a GPS-tracking database of 803 individuals across 57 mammal species found that movement activity in areas with high anthropogenic impact was on average less than half of that in areas with low anthropogenic impact. Reduction in movement may be attributable to an individual-behavioral effect, where individuals alter their movements relative to the anthropogenic impact, or a species occurrence effect, where certain species that exhibit long-range movement simply do not occur in areas of high anthropogenic impact. [3]. To better predict how animals are likely to respond to environmental change, it is therefore important to characterize movement patterns and in particular to infer the underlying mechanisms driving those patterns.

A growing number of studies has shown that individuals differ substantially in resource use [4, 5] and movement patterns [6–10] in many taxa. Interindividual variation in resource use can be related to resource availability and seasonally, thus intraspecific variation level may not be fixed over time. For instance, the scarcity of resources can lead to increased intraspecific competition and increased interindividual variation [11], [12]. Although many studies have demonstrated that individuals vary in movement patterns and habitat use, it has remained less clear what mechanisms explain this variation.

At a proximate level, features that describe movement behavior (or movement syndromes) might potentially be related to morphology (e.g., wing morphology; [13, 14]), have a behavioral basis, or a combination of both. Assuming they have a behavioral basis [15, 16] it is unclear at this point if syndromes are learned (e.g., matrilineal cultural transmission; [17]) or have a genetic basis. Personality traits such as shyness-boldness, exploration-avoidance, activity, sociability and aggressiveness may be heritable, and may have important consequences for several ecological and evolutionary processes at the population (e.g. individual movement, gene flow) and community levels (e.g., individual variation in some sets of correlated personality and morphological traits may be viewed as functional sub-categories in the organization of communities; [18]).

In heterogeneous landscapes, where animals move around to seek shelter and resources needed for their reproduction and survival, landscape structure directly affects the realized movement patterns [19]. For example, in patchy landscapes, individuals typically move fast and in a directed manner when moving among the habitat patches, whereas their movements are slow and tortuous when moving within the patches [20]. In heterogeneous landscapes, individual preferences for food resources and habitat types can interact with landscape features, generating complex movement patterns [21]. Inferring the underlying mechanisms behind such interactions can be challenging, as it requires an appropriate combination of relevant data and analytical tools [22]: spatially explicit data on both animal movements and on the availability of the relevant food resources, as well as analytical tools that capture how variation in resource availability influences individual-specific movement behavior and data on the animal’s state at different times (disease, injuries, hunger-level).

The frugivore species *Sturnira lilium* (family Phyllostomidae) is one of the most abundant and widespread bats in the Atlantic Forest [23], exhibiting a strong preference for fruits of the genus *Solanum*, followed by *Piper* and to a smaller degree *Cecropia*, *Ficus* and *Vismia* [24, 25]. In a previous study on a population of *S. lilium* inhabiting a fragmented landscape of Brazilian savanna (Cerrado), we found that different individual bats foraged more often in different geographical locations, but whether individuals have different habitat preferences or movement behavior remain to be tested [26].

Natural forest environments such as the Atlantic Forest are increasingly impacted by agricultural, pasture and human expansion (70 % of the Brazilian population live along the Brazilian Atlantic coast; [27]), most of the remaining forest being distributed in small-sized and isolated fragments immersed in anthropogenic matrix [28]. There is evidence for a fragmentation threshold for bat richness, i.e. that the number of bat species strongly depends on the forest cover (%) at the landscape level. Furthermore, studies indicate that resource use correlates with response to fragmentation: frugivorous species are most tolerant to disturbance, being abundantly present also at forest edges, secondary forests and forestry (i.e. commercial *Eucalyptus* plantations) with dense understory with many chiropterochoric plants, such as *Cecropia*, *Piper* and *Solanum* [29, 30]. This suggests that frugivorous bats can to some extent adapt to environmental changes, at least over the short term. Although *S. lilium* can also utilize disturbed areas, it needs access to areas of forest, as it needs tall trees for roosting and understory vegetation for foraging [31, 32].

Here, we investigated how the movement patterns of *S. lilium* individuals inhabiting a heterogeneous landscape respond to landscape features and food resources. We hypothesized that there is correlation between statistics that describe individual habitat preferences and movement activity, so that the variation among individuals can be summarized as low-dimensional behavioral syndromes [33]. To test these hypotheses we acquired mark-recapture data based on radio telemetry, and analyzed the data using the heterogeneous-space diffusion model introduced by [34] and [35].

## Materials and methods

### Study area

**Fig. 1:**
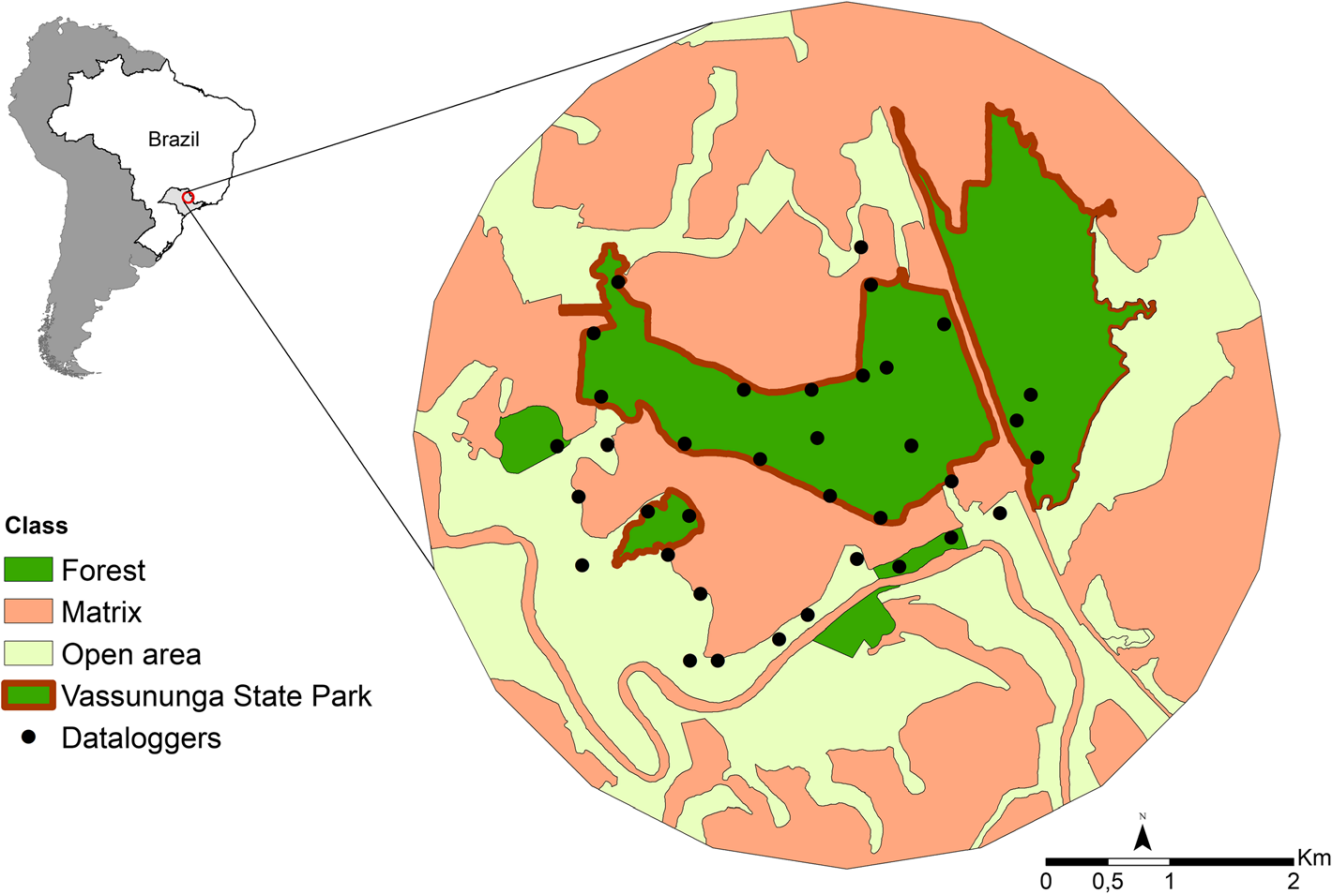
Study site: Vassununga State Park and its surroundings, where movements of *Sturnira lilium* bats were recorded in São Paulo state, Brazil

The Vassununga State Park (Parque Estadual de Vassununga - PEV) is a state nature reserve with area of c.a. 2070 ha, divided into six disjoint fragments, all of which are located in the city of Santa Rita do Passa Quatro, state of São Paulo (21º 42’ 37” S, 47º 28’ 41” W). The main vegetation types of the PEV is semi-deciduous forest (Atlantic Forest; 859 ha) and Cerrado (1,213 ha) –Brazilian savanna – surrounded by 2,776 ha of sugar cane and 1,960 ha of natural vegetation composed mainly by different physiognomy of Cerrado and riparian forest (Fig. 1).

### Bat sampling

*Sturnira lilium* bats were monitored during the wet season from September to November 2016. Bats were caught using mist nets. For each captured bat, we measured its forearm with a caliper (0.01 mm precision) and its weight with a portable dynamometer (0.1 g precision) – this information contributed in species identification in the field. Captured bats were identified by a combination of taxonomic keys [36, 37]. Twenty-seven adult individuals of *S. lilium* were selected for radio telemetry. We classified the individuals as adults or juveniles based on the degree of ossification of the epiphyses of the phalanges of the wings [38], and selected only adult individuals to avoid ontogenetic effects [39]. We included both males (N = 23) and females (N = 4) to be able to ask if there is a systematic variation between sex. In order to capture possible variability of habitat preference and food preference within the population, we sampled individuals both inside the forest and in the open areas (Fig. 1).

### Radio telemetry

We used the *Axabixo* equipment developed by *Trapa-camera* ®. The *Axabixo* device is a terrestrial radio telemetry system with digital encoding that operates both at the VHF frequency of 173,225 MHz and at the UHF frequency of 433,920 MHz (http://www.trapacamera.com.br/indexbixo.htm). The datalogger (i.e., the receiver) has an omnidirectional antenna, with range about 300 m in open areas, and it identifies the bat individual through a transmitter-specific digital code. We utilized thirty-six dataloggers (Fig. 1) that continuously recorded transmitter signals, storing information on date, time, transmitter identity and signal strength. Radio transmitters were attached to the back of the bats using veterinary glue (Vetbond). The weight of the transmitter was at most 5 % of the weight of the bat individual.

### Landscape analysis

We mapped the landscape within a 4.5 km radius buffer with the centroid located in the PEV center. We made this choice based on our previous unpublished study on *S. lilium*, in which we recorded a maximum flight distance of 6 km. The mapping was made by manual digitalization and visual interpretation using high resolution satellite images (Open Layers Plugin Google Satellite at Quantum Gis 1.8) at the 1:5,000 scale. The following cover classes were mapped: semi-deciduous forest, open natural vegetation, sugar cane, agriculture, forestry, pasture, rural facilities and water. However, for the diffusion model, we reclassified the landscape into classes that we expected to be relevant for modelling bat movements: forest (18 % within the 4.5 km radius buffer; consisting of the PEV), open area (31 %; consisting of regeneration areas and natural open physiognomies such as gramineous-woody savanna, with a predominance of pioneer plants and shrubs) and matrix (51 %; mainly sugarcane, water and highways).

We computed a set of eight landscape attributes for each location of dataloggers: the proportions of forest cover (FC), open area cover (OA) and matrix cover (MA) in a circular buffer with 200 m radius, the spatial heterogeneity (HE) of the buffer calculated by the Shannon’s landscape diversity index; distance of the datalogger to the nearest forest fragment (NN), and estimated numbers of ripe fruits of trees in the genera *Piper* (PI), *Solanum* (SO) and *Cecropia* (CE). These trees provide the main resources consumed by S. *lilium* [24, 25]. We chose a 200 m radius buffer because it was the maximum signal range of the transmitters in forest areas. The landscape was mapped using Quantum Gis 1.8 (QGIS Development Team) and ArcGis 10.5. The landscape metrics were calculated on the extension V-Late.

### Fruit availability

For estimating the number of fruits, we also used a 200 m radius buffer around each datalogger. Inside each buffer, we first estimated the number of fruits per branch as the average count over 10 individual trees. The trees were selected randomly from the point where the datalogger was located. Second, we defined four perpendicular transects and counted the number of branches of each plant species along each transect. Finally, we multiplied the number of branches by the average number of fruits per branch.

### Fitting the heterogeneous-space diffusion model

We fitted to the bat detection data the heterogeneous-space diffusion model introduced by [34] and [40], and extended to account for variation among species by [35]. In this study, we consider a single species, but utilize the structure of the Joint Species Movement Modeling (JSMM) framework [35] to model variation among individuals. The diffusion model (which is technically a partial differential equation, see [34]) accounts for habitat-specific variation in movement and mortality rates, and for habitat selection at edges between habitat types. Prior to the analyses, we triangulated the landscape (as needed to fit the diffusion model to data; [34]) classified into the three habitat types: forests ($h=1$), matrix ($h=2$), and open areas ($h=3$) – this step was done using the software Mapper [40]. The movement model contains seven parameters to be estimated for each individual $i$: (a) three habitat-specific movement rates (the diffusion parameters ${D}_i^h$ for each habitat type $h$); (b) two habitat preference parameters ${k}_i^h$ (relative preferences to open areas and to matrix, normalized to 1 for forests); (c) mortality parameter ${m}_i$ (assumed to be the same for all habitat types) and (d) detection probability ${q}_i$ (assumed to be the same for all habitat types). We note that in the present study the mortality parameters are more likely to represent the death of the battery of the tracking equipment rather than the death of the individual bat.

The detection probability ${q}_i$ measures the probability of observing the individual by a datalogger conditional on the individual being within the detection area [34]. We defined for each datalogger a detection area based on a field experiment where we examined from which locations the datalogger was able to detect the tracking equipment. We then doubled the sizes of these areas to account for small-scale bat movements that may bring the individual to the detection area during one-time step when the individual is nearby it. We note that the detection probabilities (${q}_i\Big)$ are likely to be similar for each individual, even if the devices may somewhat vary in their technical quality. We chose to include variation among individuals in detection probability partially because it allowed us to utilize the modelling framework of [40].

The ${n}_p=7$ individual-specific movement parameters can be described by the vector

1 $$ {\varTheta}_{i\bullet}\kern0.5em =\left(\log \left({D}_i^1\right)\kern1em \log \left({D}_i^2\right)\kern1em \log \left({D}_i^3\right)\kern1em \log \left({k}_i^2\right)\kern1em \log \left({k}_i^3\right)\kern1em \log \left({m}_i\right)\kern1em \mathrm{logit}\left({q}_i\right)\right) $$

We computed the likelihood $p\left({\varvec{y}}_{i}|{\varvec{\Theta }}_{i\bullet }\right)$ for observing the movement data ${\varvec{y}}_{i}$ using the finite-element scheme to solve the time-evolution of the probability density for the individuals’ location, as described by [34] and implemented for the case of variation among species (here, ${n}_{i}$ individuals) by the JSMM [35]. The data are in continuous-time, but the implementation of the diffusion model requires one to define discrete time intervals at which the detections are attempted, which we set to 12 h intervals.

The JSMM estimates species- and community-level movement parameters as a function of species traits and their phylogenetic relationships. Here, we model the movement activity of bats dependent on individuals’ weight and sex. We combine the individual-specific parameters into the ${n}_{i}\times {n}_{p}$ matrix $\varvec{\Theta }$, and then model the ${n}_{i}{n}_{p}\times 1$ vector $\varvec{\uptheta }=\text{v}\text{e}\text{c}\left(\varvec{\Theta }\right)$ using a multivariate normal distribution

2 $$\varvec\uptheta \sim\text{}{N}\left(\mathbf{m}, \varvec{\Sigma }\otimes {{\mathbf{I}}_{n}}_{i}\right)$$

Here the ${n}_{p}\times {n}_{p}$ variance-covariance matrix $\varvec{\Sigma }$ models the individual-specific deviations from the expectations based on the traits, ${{\mathbf{I}}_{n}}_{i}$ is the ${n}_{i}\times {n}_{i}$ identity matrix, and $\otimes$ is the Kronecker (outer) product. The vector $\mathbf{m}=\text{v}\text{e}\text{c}\left(\mathbf{M}\right)$ is the vectorized version of the ${n}_{i}\times {n}_{p}$ matrix $\mathbf{M}$, with the expected movement parameters based on individuals’ sex, and weight. We model the matrix elements ${m}_{ip}$ for each individual $i$ and parameter $p$ as

3 $$ {m}_{ip}={\sum}_a{t}_{ia}{\zeta}_{ap} $$

where $a$ is the index for ${n}_a=3$ traits (sex and weight, as well as intercept modelling the overall mean), and ${t}_{ia}$ is the trait $a$ for bat $i$. The parameter ${\zeta}_{ap}$ measures the effect of traits (sex and weight) on parameter $p$. We log-transformed the weight values, and converted sex categories into a binary variable (1 for males and 0 for females).

We ran a Monte Carlo Markov Chain (MCMC) algorithm to estimate the parameters, first for 50,000 iterations, during which we adapted the proposal distributions of $\varvec{\Theta }$ to achieve optimal mixing, and then sampled the posteriors by running the MCMC algorithm further by 350,000 iterations. We replicated the MCMC sampling for 3 independent chains, and assessed the convergence of the MCMC algorithm using the Gelman-Rubin convergence statistic [41] with the package CODA [42].

### Deriving ecological inference from the diffusion model

To assess model fit, we simulated movement tracks, using individual specific parameter values sampled from the posterior distribution. To make real and simulated data as comparable as possible, we released each virtual bat in the location where a real bat was first observed, and we assumed the same spatial distribution of dataloggers as was used in the actual field study. We compared 1000 replicates of simulated data to observed data with sixteen statistics (S1-S16) that we considered relevant indicators of movement behavior. The statistics S1-S8 are the mean values of the eight landscape indices describe above (FC, OA, MA, HE, NN, PI, SO and CE), averaged over the locations where the individual was observed. The remaining statistics are the fraction of observations in forest (S9) and open habitats (S10), the proportion of consecutive observations in which the bat changed from one habitat type to another habitat type (S11), the mean distance between consecutive observations (S12), the distance between the first and the last observation (S13), the number of distinct receivers in which the bat was observed (S14), the proportion of observations in the receiver from which there were most observations (S15), and the proportion of time-steps from first to last observation in which the bat was detected (S16, Table 1). To validate the structural assumptions of the diffusion model, we averaged the statistics S1-S16 over the individuals and compared the statistics for real data to posterior mean and 95 % credible intervals [CI] derived from the simulations.

**Table 1:** Statistics and description of the movement of *Sturnira lilium* bats inhabiting a heterogeneous landscape in Brazil

Statistic	description
S1	Mean forest cover
S2	Mean open area cover
S3	Mean matrix cover
S4	Mean heterogeneity
S5	Mean distance to nearest forest
S6	Mean number of *Piper* fruits
S7	Mean number of *Solanum* fruits
S8	Mean number of *Cecropia* fruits
S9	Proportion of observations in forest
S10	Proportion of observations in open area
S11	Proportion of habitat change
S12	Mean distance between observations
S13	Distance from first to last observation
S14	Number of distinct receivers
S15	Proportion in most frequent receiver
S16	Proportion of time-steps within observations

We assessed the presence of characteristic movement syndromes by examining if the variation among individuals showed correlated patterns in their movement characteristics. As movement characteristics, we used the posterior means of the individual-specific parameters of the movement model (diffusion rates and habitat preferences) and the residual statistics S1-S16, i.e. the difference between observation and the posterior mean prediction by the diffusion model. The reason for including the residual rather than the raw statistics was that they measure deviations of the movement characteristics S1-S16 from the null expectation based on the estimated movement parameters at the population scale. To make statistics with different units comparable, we normalized them to zero mean and unit variance over the individuals. We performed a Principal Component Analysis (PCA) to summarize the information contained in the continuous multivariate movement data considering the 23 residual statistics as the variables, and a k-means clustering to split the individuals into a set of 4 clusters. The optimal number of clusters (4) was determined by the Average Silhouette Method, which determines how well each object lies within its cluster [43]. We performed the PCA analysis using the prcomp function and the k-means analysis in R [44]. We used the factoextra R package to help in the interpretation and visualization of PCA and clustering analysis.

## Results

**Fig. 2:**
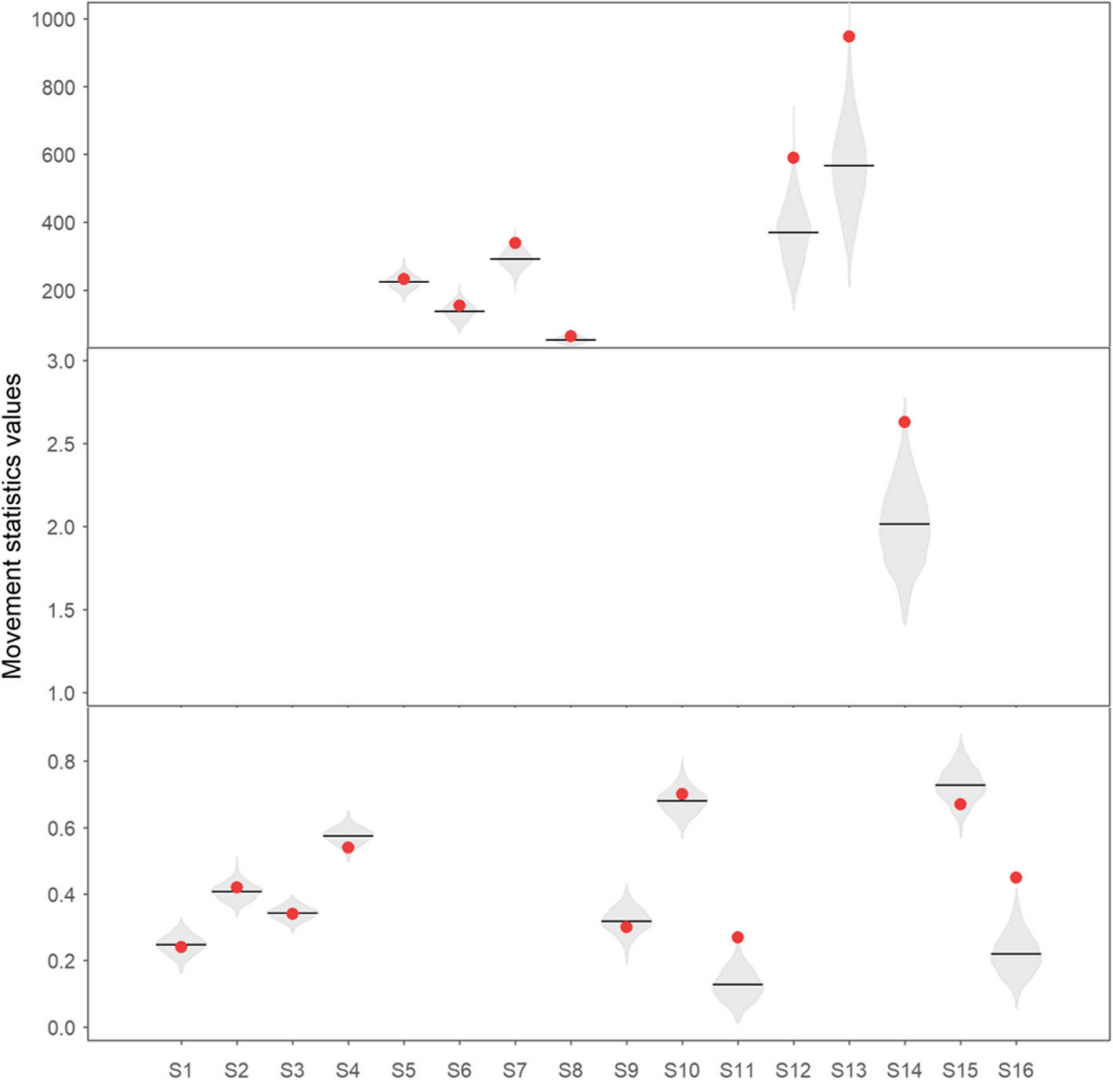
A comparison of population-level movement statistics between real and simulated data. The black lines are the statistics mean values for the simulated data, and the red dots are the statistic values for the real data. The shadowed areas show the distribution of the summary of 1000 simulations

We detected 7607 records (mean ± standard deviation = 271.7 ± 411.9, *n* = 27) and the mean number of days between first and last record per individual was 6 (+-6, *n* = 27). At the species level, habitat preference order of the bats was greater for open areas than for forest or matrix, bats preferred locations with high availability of *Solanum* fruits and bats changed between forest and open area habitats frequently. At the individual level, we detected four classes of individuals: (1) average individuals, (2) forest specialists, (3) explorers which prefer *Piper*, and (4) open area specialists which prefer *Solanum* and *Cecropia* (Fig. 2).

### Parameter estimates

The posterior means of the diffusion model parameters for the studied population are shown in Table 2 (see all the parameter estimates and the convergence diagnostics in Supporting Table S5). The model indicates that the habitat preference order of the bats was greater for open areas than for forest or matrix, and that movement rates were not related to habitat preferences so that the movement had similar diffusion rates in all habitats. The mean lifetime of bats (or batteries), which can be computed as the reciprocal of the mortality rate, was ca. 13 days. The estimated detection probability was very close to one, indicating that bats that were close to the vicinity of a datalogger were very likely to be detected by the equipment.

**Table 2:** The posterior means and 95 % credible intervals of the diffusion model parameters, for the studied bat population. The habitat preference parameters ${k}^h$are unitless. The unit of diffusion ${D}^h$ is m^2^/day. The unit of mortality $m$ is 1/day

Parameter	Mean		0.025 quantile	0.975 quantile
Preference for forests (${k}^1$)	1.00		1.00	1.00
Preference for matrices (${k}^2$)	1.13		0.26	3.76
Preference for open areas (${k}^3$)	101.17		15.00	226.40
Diffusion in forest (${D}^1$)	337,419.65		180,033.24	691,128.32
Diffusion in matrix (${D}^2$)	332,964.35		135,208.25	757,394.96
Diffusion in open areas (${D}^3$)	200,588.34		79,495.71	642,622.82
Mortality ($m$)	0.15		0.05	0.67
Detection probability ($q$)	0.86		0.68	0.96

### Structural model validation

**Fig. 3:**
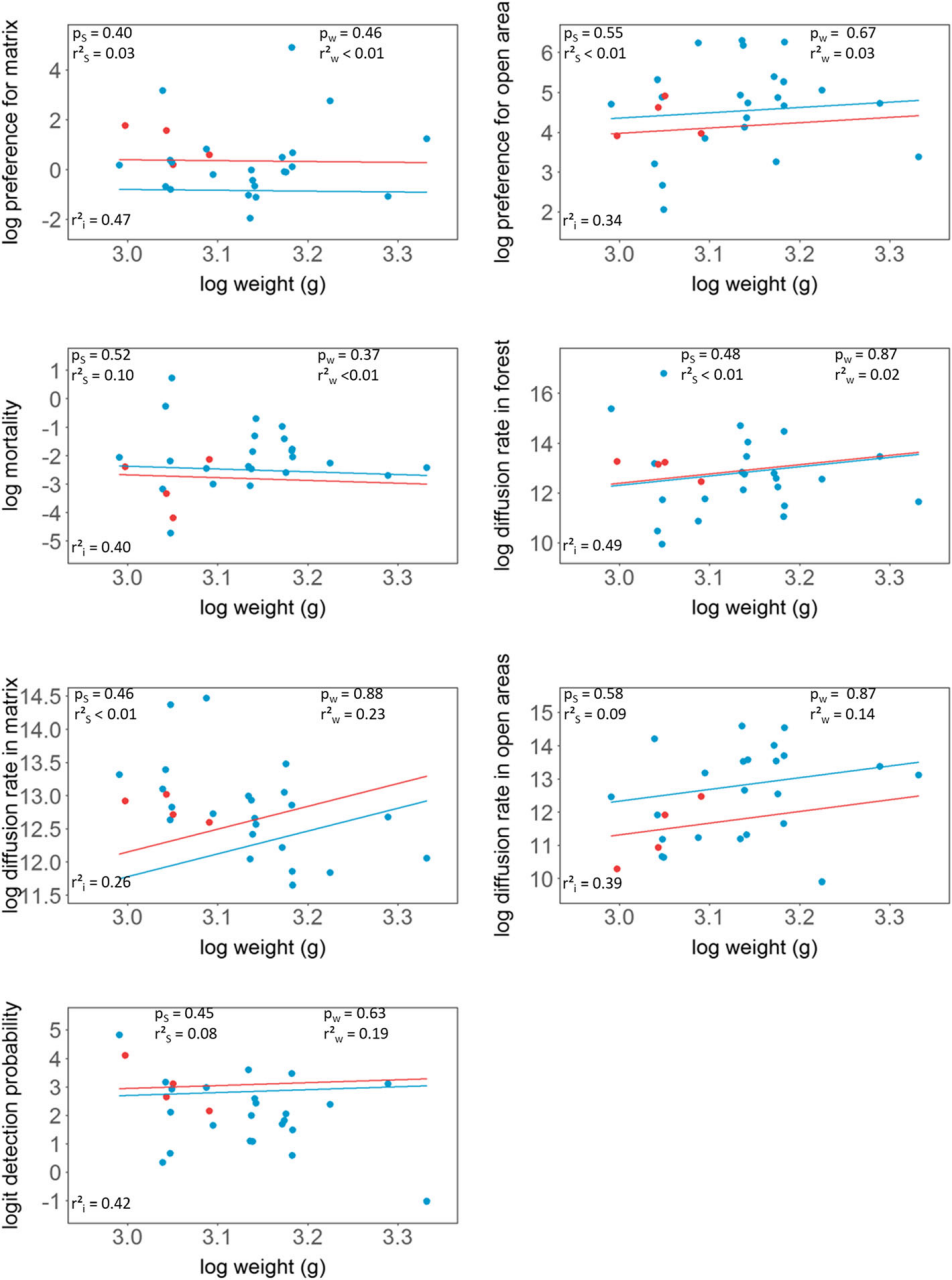
Individual-specific parameters estimates. The dots show the posterior mean for each male (blue) and female bat (red). The lines show the expected posterior means based on bat weight and sex – females (red) and males (blue). The posterior probability of weight having a positive effect on the movement parameter is denoted by p_w_. The posterior probability of males having a larger movement parameter than females is denoted by p_s_. The proportion of variation of each movement parameter explained by weight and sex are denoted by r²_w_ and r²_s_, respectively. We show for each movement parameter the amount of variation not explained by bat weight and sex (r²_i_)

The simulated data and the real data matched well in terms of the statistics S1-S6 and S8-S10 describing the distribution of the bats with respect to habitat types (Fig. 3). This is to be expected, as the diffusion model accounted explicitly for the habitat composition of the landscape. Deviations between simulated and observed data were minor also for statistics that were not directly fitted in the diffusion model. For example, the simulated data slightly underestimated the amount of *Solanum* fruits in the locations where the bats visited (S7), meaning that the bats preferred locations with high availability of fruits more than predicted by the diffusion model that does not explicitly account for fruit availability. As a second example, bats changed between habitat types more frequently than proposed by the simulated data (S11), indicating that they alternate between forest and open area habitats more often than predicted by the diffusion model that is based on a random walk assumption. As a third example, simulated data somewhat underestimated the proportion of times the bats were detected (S16), suggesting that the delineated detection area was too small for modelling the detection process of bats at this time scale.

### Individual variation in movement behavior

**Fig. 4:**
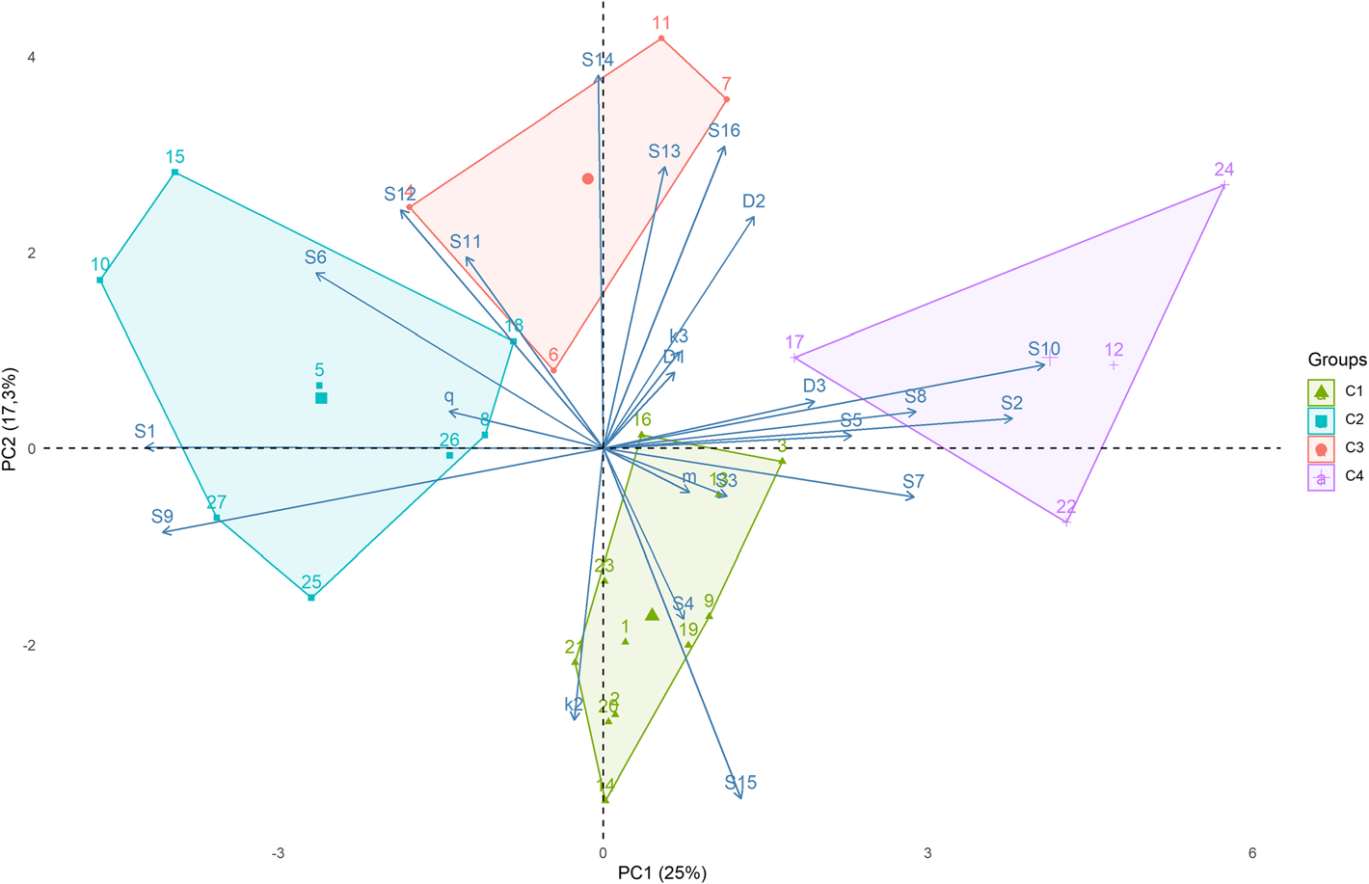
Results of a k-mean analysis aimed at detecting behavioral syndromes of Sturnira lilium. Numbers represent individuals. Each color represents the syndrome that each individual belongs, determined by k-means analysis. The largest axis of variation PC1 (25 % of statistics variance) separates mainly habitat use, increasing PC1 meaning increasing use of forests, and PC2 (17.3 %) separates mainly long-term movement activity, increasing PC2 meaning exploratory (as compared to stationary) behavior

The sex and the weight of the bats explained less than 10 % of variation in posterior movement parameters, except for the mortality rate (Fig. 4). Despite the overall little amount of variation explained by bats’ traits, we found evidence that larger bats have larger diffusion rates in open areas, and that male bats have a higher mortality rate (probably related to battery life). For all other movement parameters, weight and sex explained only little of the variation among individuals (Fig. 4).

We identified four main movement syndromes according to a cluster analysis using the correlation among the statistics and a PCA analysis (Fig. 2; Table 3, Additional file S1 and S4. However, it is important to note that the variation between individuals can be gradual rather than very well-defined clusters. We used a k-mean analysis to divided bats into four classes: (1) average individuals, (2) forest specialists, (3) explorers which prefer *Piper*, and (4) open area specialists which prefer *Solanum* and *Cecropia* (Table 3; Fig. 2). Figures with the movement of each individual can be seen in Additional file S2, a synthesis of the data obtained from each individual is provided in Additional file S3.

**Table 3:** Central values of statistics for each group of movement syndrome, classified with K-means clustering.

**ClusterID**	**S1**	**S2**	**S3**	**S4**	**S5**	**S6**	**S7**	**S8**	**S9**	**S10**	**S11**	**S12**	**S13**	**S14**	**S15**	**S16**	**K2**	**K3**	**m**	**D1**	**D2**	**D3**	**q**
**1**	-0.18	0.02	0.27	0.16	0.28	-0.22	0.11	0.08	-0.02	0.02	-0.54	-0.67	-0.61	-0.74	0.78	-0.51	0.33	-0.23	0.37	-0.16	-0.42	-0.07	-0.13
**2**	1.09	-0.97	-0.31	0.06	-1.06	0.37	-0.54	-0.41	1.14	-1.14	0.51	0.65	0.25	0.26	-0.51	-0.07	0.12	0.16	-0.58	0.22	-0.06	-0.14	0.17
**3**	-0.16	0.35	-0.29	-0.95	0.96	1.24	-0.46	-0.57	-0.66	0.66	0.31	0.47	0.49	1.35	-1.05	1.1	-1.07	-0.15	-0.17	-0.49	0.28	-0.78	0.86
**4**	-1.53	1.53	0.16	0.39	0.38	-1.38	1.24	1.16	-1.55	1.55	0.15	0.08	0.69	0.18	-0.07	0.45	-0.09	0.45	0.33	0.48	0.98	1.25	-0.83

## Discussion

Our results indicate that individuals in a population of a common frugivorous bat species vary in how their movement depends on landscape elements. Importantly, we show that variation in different aspects of movement behavior can be summarized in terms of behavioral movement syndromes. Kerches-Rogeri et al. (2020) demonstrated that different individual bats foraged more often in different geographical locations regardless of habitat type. In our present study, we go one step further and demonstrate that individuals have different habitat preferences and movement characteristics.

The fact that we found a weak effect of sex on movement can be related to the small sample size of females. However, we consider that this deserves future studies because the shape of the wings may be different between the sexes because females carry extra weight during pregnancy and wing shape must keep aerodynamics of flight [45], but we do not know whether movement and habitat selection are related to differences in wing morphology.

Regarding weight, larger bats tended to have high movement rate in open areas. A similar trend of body size effect in space use can be found in interspecific studies at other taxa [3, 46, 47], but there are only indications of this trend among bats (e.g. [48, 49]). Our results thus suggest that also intraspecific variation in body size is relevant for understanding how bats move within a landscape.

Some studies indicate that *S. lilium* is preferentially associated with secondary forest (e.g. [30], [50]), whereas other studies indicate an association with more mature forests [51]. Our results may help to elucidate these descriptions, by showing that a single population may contain individuals with preferences for different habitat types. Because the degree of inter-individual variation may vary among populations [4], the presence of syndromes may be population-specific rather than universal, since each population faces different environments and conditions, e.g. resource availability and predation pressure [52]. It is possible that some populations are behaviorally monomorphic (either specializing in open or forest habitats) and others (e.g., the population studied here) are polymorphic, which would help to explain these apparent conflicting findings.

Intraspecific variation can affect important processes to the survival of the species. The niche overlap among individuals can increase or decrease competition, since variation between individuals of the same species can decrease competition between conspecifics, but increase competition with other species [11]. In another scenario, the variation between individuals can also be a relief for inter-specific competition, since even if the overlap between the niche of other species increases, this impact can be reduced, because only a subgroup of individuals in each species are affected.

Intraspecific variation can also be important for ecosystem processes performed by the species. Because *S. lilium* is an important seed disperser and a “fragment connector” for many plant species in the Neotropics [53, 54], the presence of movement syndromes may have implications for this ecological service. The importance of variation within a population of dispersers may be related to Jensen`s inequality mechanism, in which when an ecological interaction depends nonlinearly on a specific characteristic, the variation around the mean of that characteristic can change the average strength the interaction [11, 55]. If a population has different subsets of individuals with respect to the partition of landscape use, it may mean that the real connectivity of the landscape by the bats may be different from the connectivity provided by a population formed by average individuals.

Individuals with different syndromes are likely to interact with different plant species and disperse seeds to different areas with different efficiencies, suggesting that they might vary in their quality as dispersers [55, 56]. Individuals using forest areas, for instance, are likely to be effective dispersers of *Piper* seeds, whereas individuals using open areas are likely to be effective dispersers of *Solanum* seeds. Understanding how inter-individual variation in movement patterns in *S. lilium* affects seed dispersal and the dynamics of the plants they disperse should be addressed in future research.

From a conservation biology point of view, the presence of movement syndromes suggests that individuals are likely to respond differently to habitat change, which may be good news, since the adaptation capacity of these bats to different natural and anthropogenic habitat may guarantee their maintenance within human-modified landscapes. Individuals specialized in open habitats might benefit from forest fragmentation, whereas those specialized in forest habitats should be negatively impacted. By the same token, restoration programs aiming at recovering only dense forest habitats may be advantageous for only a subset of the population. Besides, if individual specialization is correlated with the number of coexisting species (as predicted by the niche variation hypothesis), then protecting a highly variable population of a species may require protecting a habitat of little diversity, while reserves designed to include high interspecific biodiversity can minimize intraspecific diversity (52). Therefore, conservation efforts may benefit from considering how changes in habitat may impact individuals differently in a population.

It is important to note that our study describes movement for a short period of time (with an average battery life of 13 days), so we do not know whether bats consistently fall into a single behavioral syndrome or they switch back and forth, just as we do not know whether this is a consistent individual-level trait or is it subject to unmeasured temporal factors. One of the causes of individual specialization is intraspecific competition, which is related to the availability of resources, therefore, the same population may exhibit strong or weak niche overlap between individuals according to the availability of resources. Thus, it would be plausible to assume that the organization of bat individuals in groups is subject to seasonal variation related to the natural seasonal availability of resources.

## Conclusions

Our results indicate that individuals may vary consistently in their movement patterns, which can be considered movement syndromes. Individual preferences for elements of the landscape highlight the importance of incorporating explicitly the landscape to descriptions of animal movement. An important step in future research is to understand more mechanistically the eco-evolutionary processes driving such variation, and to account for movement syndromes in conservation efforts, e.g. related to the consequences of habitat fragmentation on seed dispersal.

## Supplementary information

Additional file 1 **S1.** The rationale behind the interpretation of the syndromes.

Additional file 2 **S2.** Individuals-movement.

Additional file 3 **S3.** Datalogger records summary.

Additional file 4 **S4.** Clusters definition and PCA output.

Additional file 5 **S5.** Parameter estimates and convergence diagnostics.

## Data Availability

Data will be available on MoveBank (https://www.movebank.org).

## Abbreviations

GPS: Global positioning system
PEV: Parque Estadual de Vassununga
FC: forest cover
OA: open area cover
MA: matrix cover
HE: spatial heterogeneity
NN: distance of the datalogger to the nearest forest fragment
PI: estimated numbers of ripe fruits of trees in the genus *Piper*
SO: estimated numbers of ripe fruits of trees in the genus *Solanum*
CE: estimated numbers of ripe fruits of trees in the genus *Cecropia*
MCMC: Monte Carlo Markov Chain
CI: credibility interval
