# Supplementary material for: Movement syndromes of a Neotropical frugivorous bat inhabiting heterogeneous landscapes in Brazil
Source: Mov Ecol. 2021 Jul 7;9:35. doi: 10.1186/s40462-021-00266-6 (PMC8262009; doi:10.1186/s40462-021-00266-6)
Supplement: Supplementary file 1 — S1. The rationale behind the interpretation of the syndromes. [file 40462_2021_266_MOESM1_ESM.docx]

Additional file S1. The rationale behind the interpretation of the syndromes.

Table S1-1. Simplification of the numerical results of the clustering (shown in Table 3). The table shows for each statistic (rows) the clusters (columns) that respond positively (+) and negatively (-), with the criteria that the group mean is at least one standard deviation above (+) or below (-) the overall mean. It also provides a keyword for each statistic to aid their quick interpretation.

|  | Description | keyword | C1 | C2 | C3 | C4 |
| --- | --- | --- | --- | --- | --- | --- |
| S1 | Mean forest cover | forest |  | + |  | - |
| S2 | Mean open area cover | open |  | - |  | + |
| S3 | Mean matrix cover | matrix |  |  |  |  |
| S4 | Mean heterogeneity | heterogeneity |  |  | - |  |
| S5 | Mean distance to nearest forest | no forest |  | - | + |  |
| S6 | Mean number of *Piper* fruits | piper |  |  | + | - |
| S7 | Mean number of *Solanum* fruits | solanum |  |  |  | + |
| S8 | Mean number of *Cecropia* fruits | cecropia |  |  |  | + |
| S9 | Proportion of observations in forest | forest |  | + |  | - |
| S10 | Proportion of observations in open area | open |  | - |  | + |
| S11 | Proportion changed habitat | many habitats |  |  |  |  |
| S12 | Mean distance between observations | local movement |  |  |  |  |
| S13 | Distance from first to last observation | large-scale movement |  |  |  |  |
| S14 | Number of distinct receivers | large-scale movement | - |  | + |  |
| S15 | Proportion in most frequent receiver | stationarity | + |  | - |  |
| S16 | Proportion of time-steps within observations | detection |  |  | + |  |
| K2 | Preference for matrices ($k^{2}$) | matrix |  |  | - |  |
| K3 | Preference for open areas ($k^{3}$) | open |  |  |  |  |
| m | mortality | mortality |  |  |  |  |
| D1 | Diffusion in forest ($D^{1}$) | movement (forest) |  |  |  |  |
| D2 | Diffusion in matrix ($D^{2}$) | movement (matrix) |  |  |  |  |
| D3 | Diffusion in open areas ($D^{3}$) | movement (open) |  |  |  | + |
| q | Detection probability ($q$) | detection |  |  |  |  |

We note that in Fig. 4 the Clusters 2 and 4 are at the extremes of PC1 and thus represent contrasting syndromes, that Clusters 3 is the extremes of PC2 and thus represent explorer syndromes, and Cluster 1 is in the middle of the ordination space and thus represents the population average (average individuals). Based on Table S1 and Fig. 4, we interpret the syndromes as follows.

Cluster 1: Average individuals

Cluster 2: Forest specialists

Cluster 3: Explorers, prefer Piper

Cluster 4: Open area specialists, prefer Solanum and Cecropia

PC1 separates mainly habitat use, increasing PC1 meaning increasing use of open areas. PC2 separates mainly long-term movement activity, increasing PC2 meaning exploratory (as compared to stationary) behavior.
