# Supplementary material for: Movement syndromes of a Neotropical frugivorous bat inhabiting heterogeneous landscapes in Brazil
Source: Mov Ecol. 2021 Jul 7;9:35. doi: 10.1186/s40462-021-00266-6 (PMC8262009; doi:10.1186/s40462-021-00266-6)

Additional file S2. Movement of the Neotropical frugivorous bat *Sturnira lilium* inhabiting heterogeneous landscapes in Brazil

The dashed line represents the trajectory displayed by each individual among the dataloggers. Different dashed line colors represent different days of monitoring. Circles represent that individual had many records in that location. It is important to note that these figures represent just how individuals use the landscape, since we consider the exact location of the dataloggers as the point of movement.
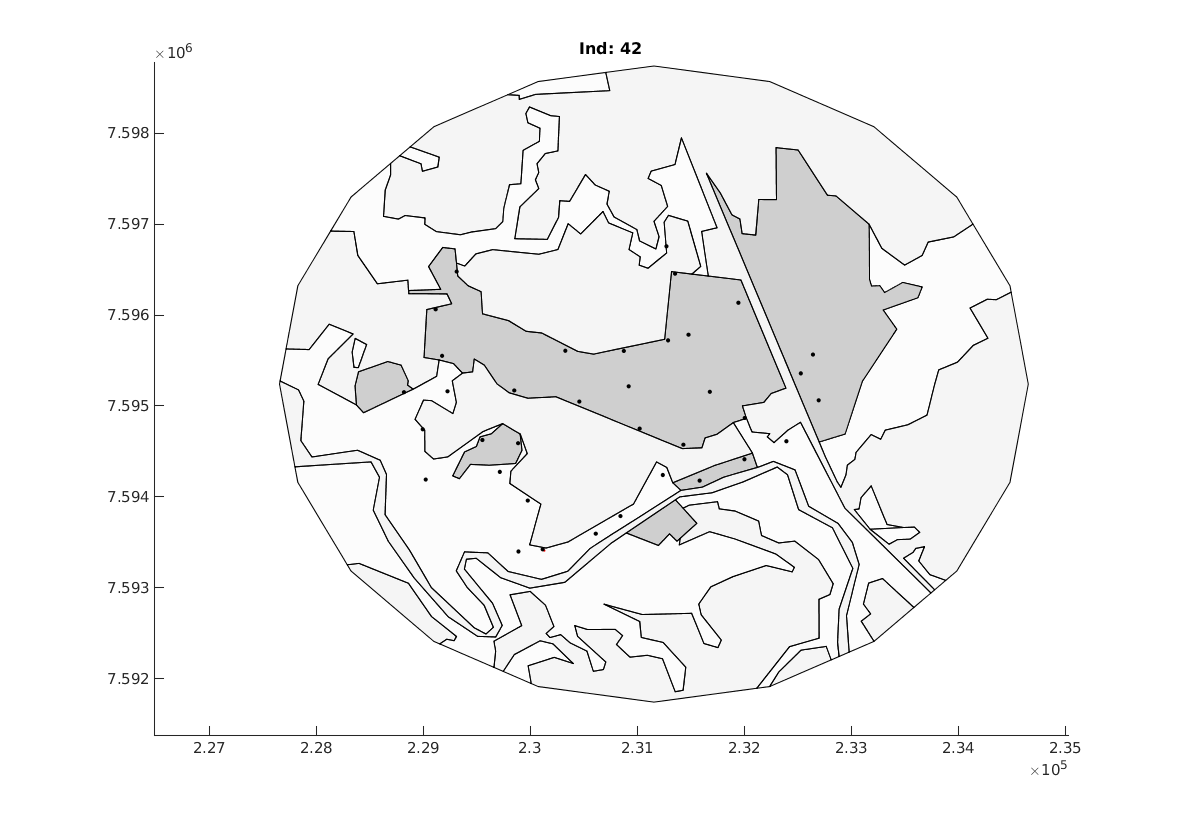

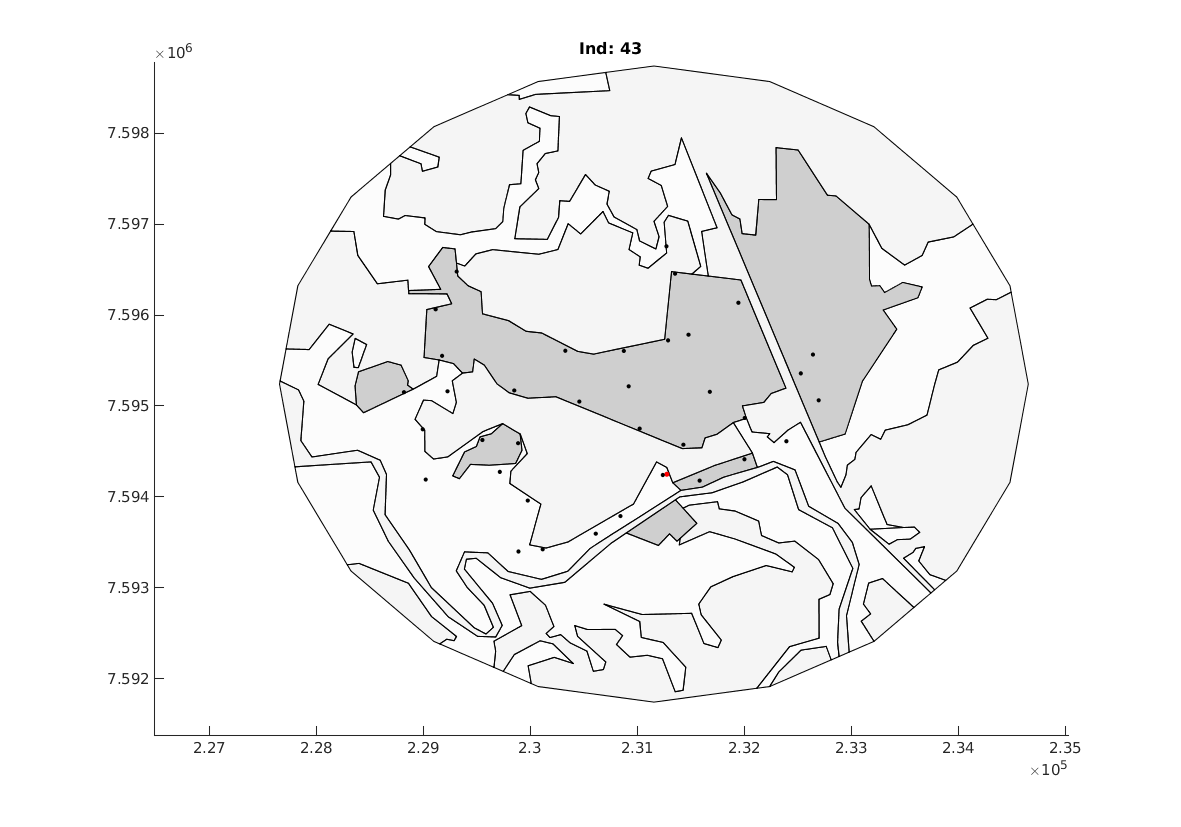

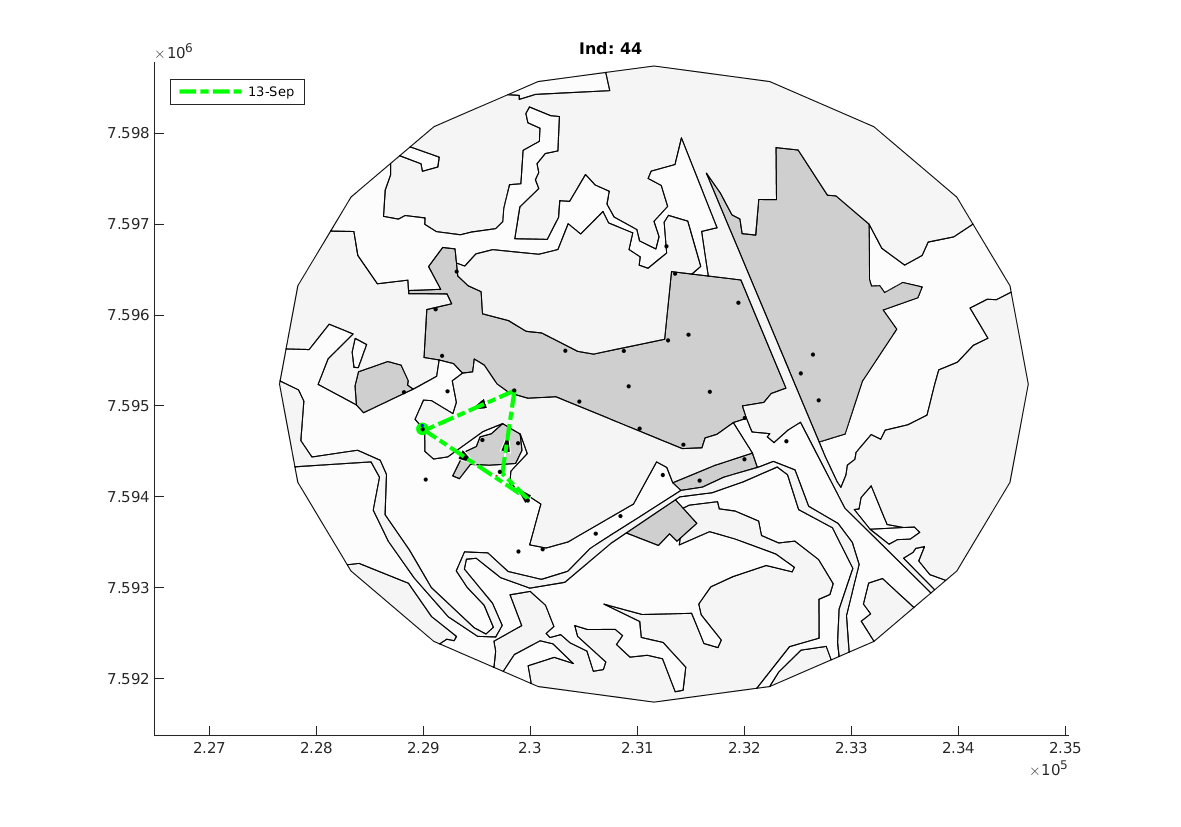

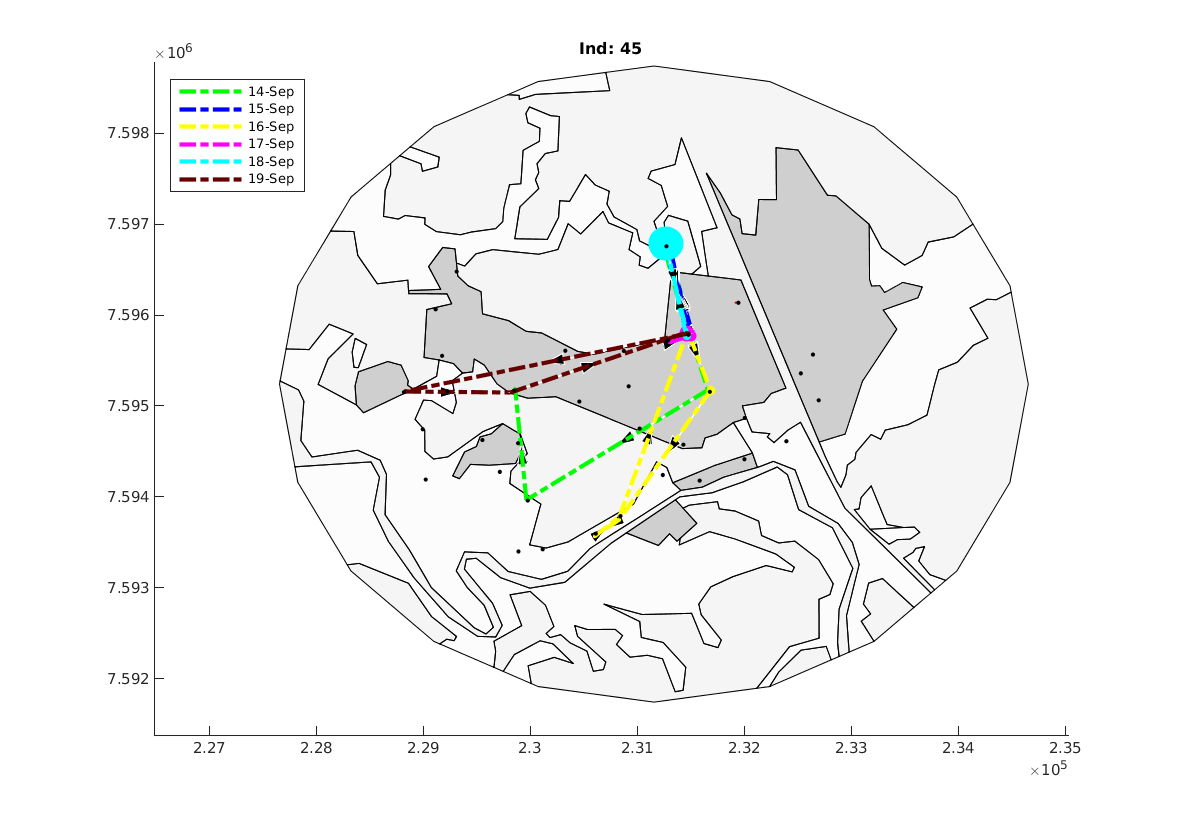

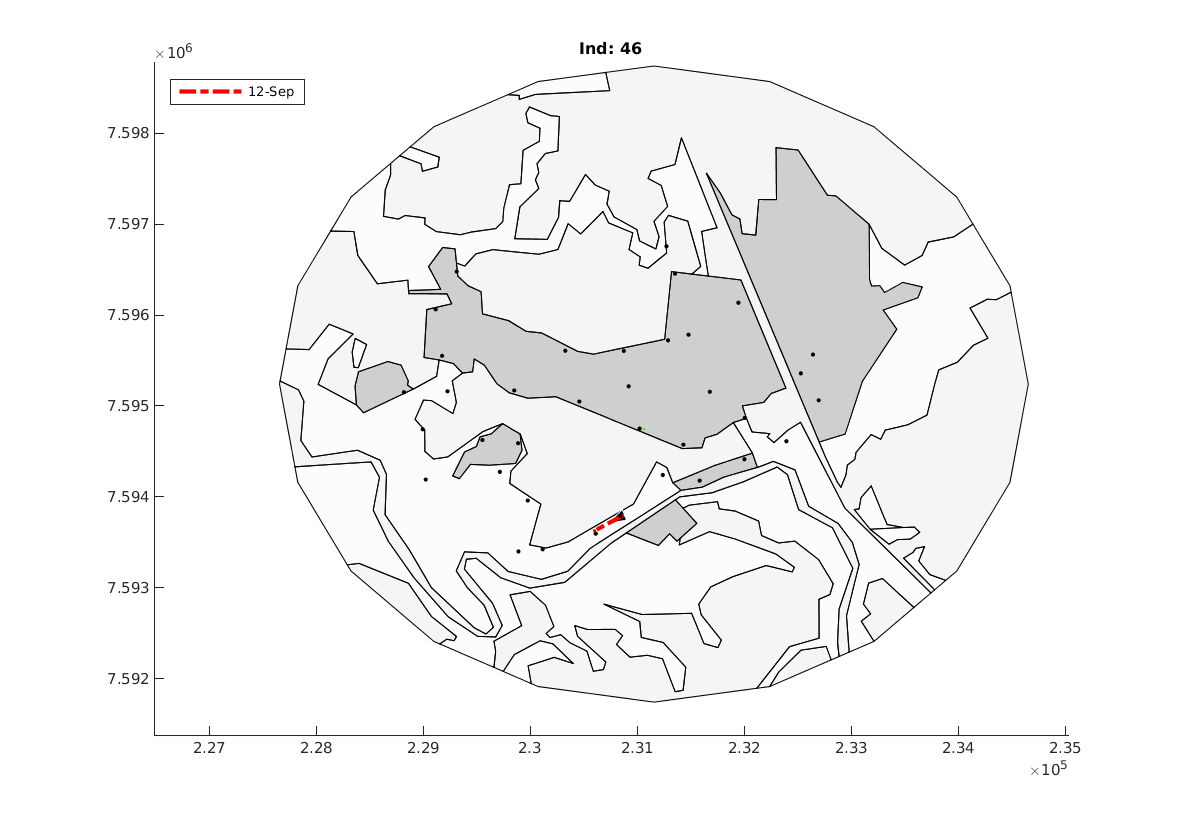

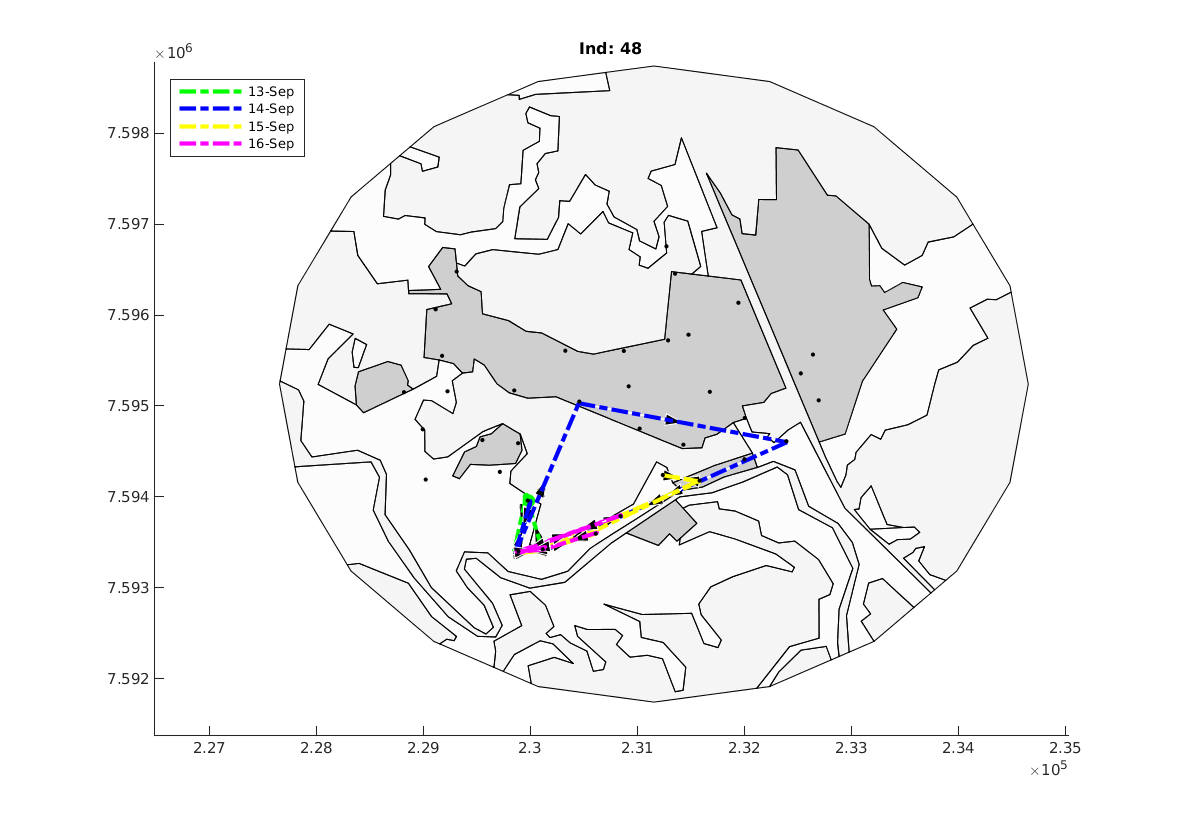

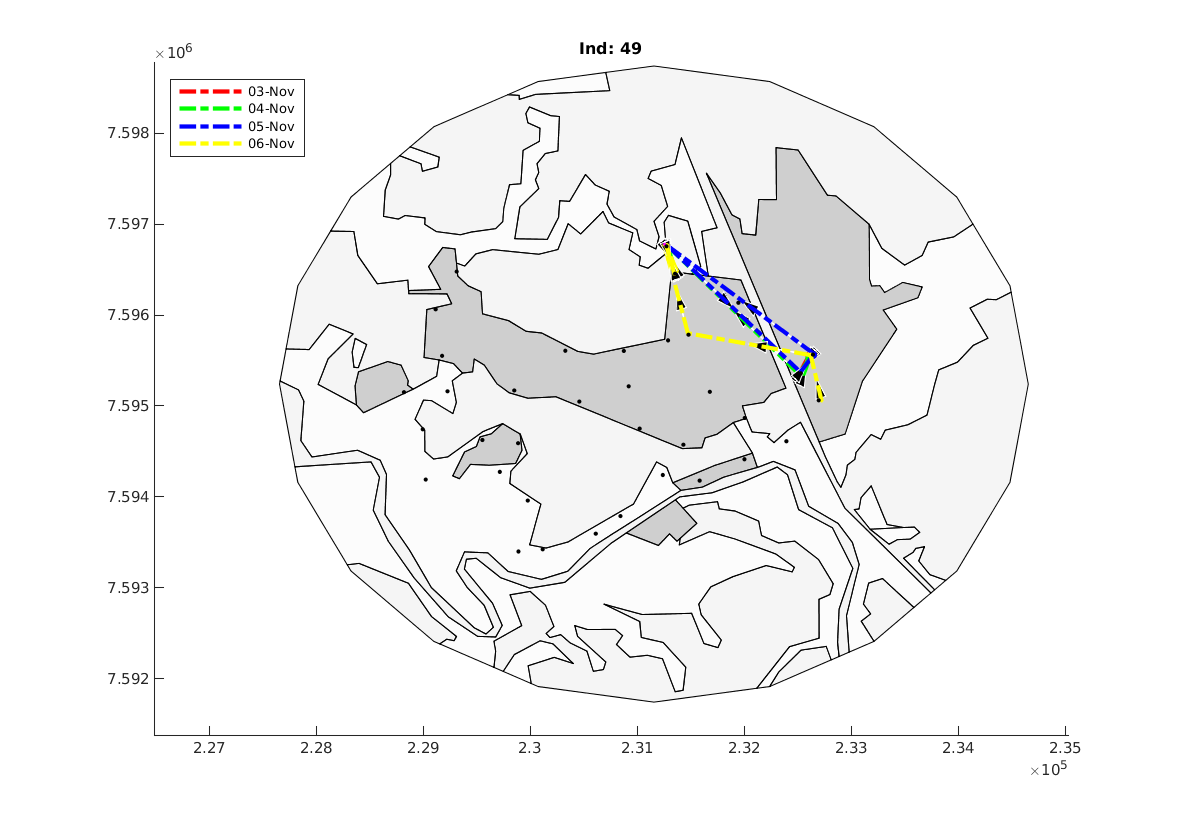

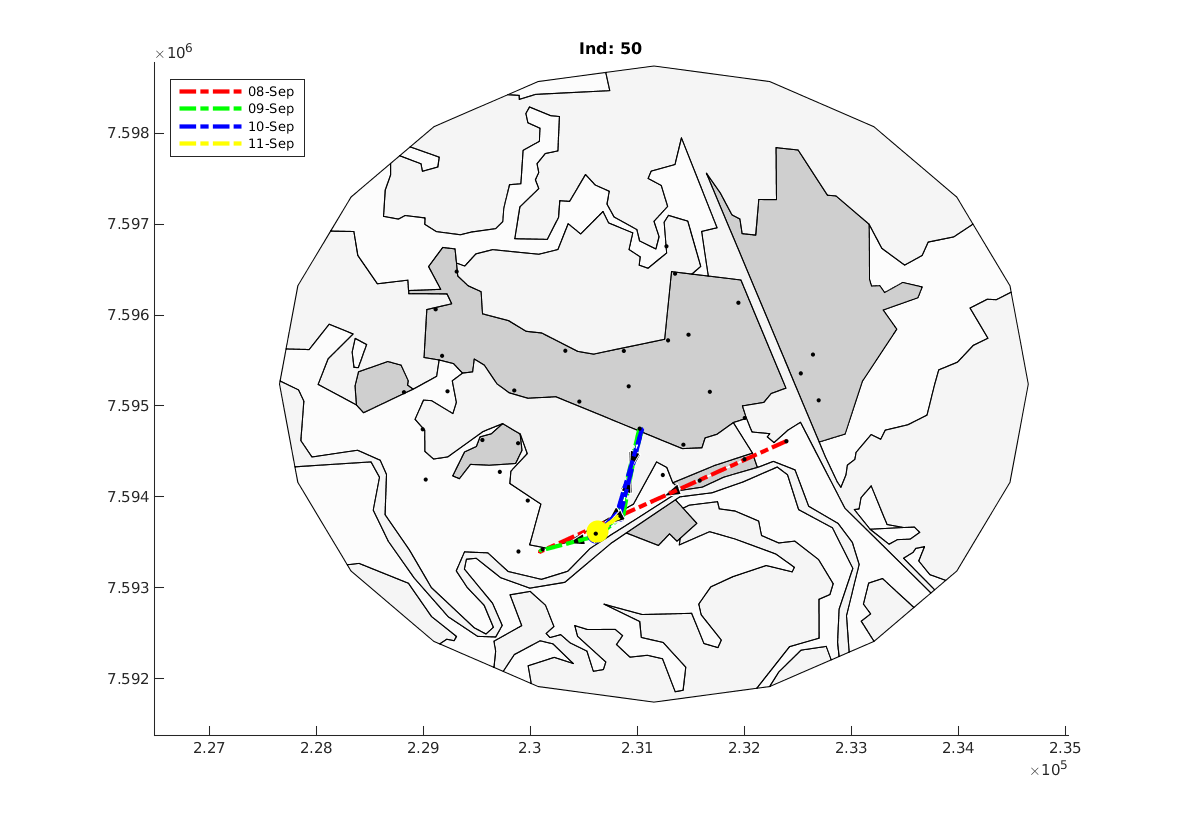

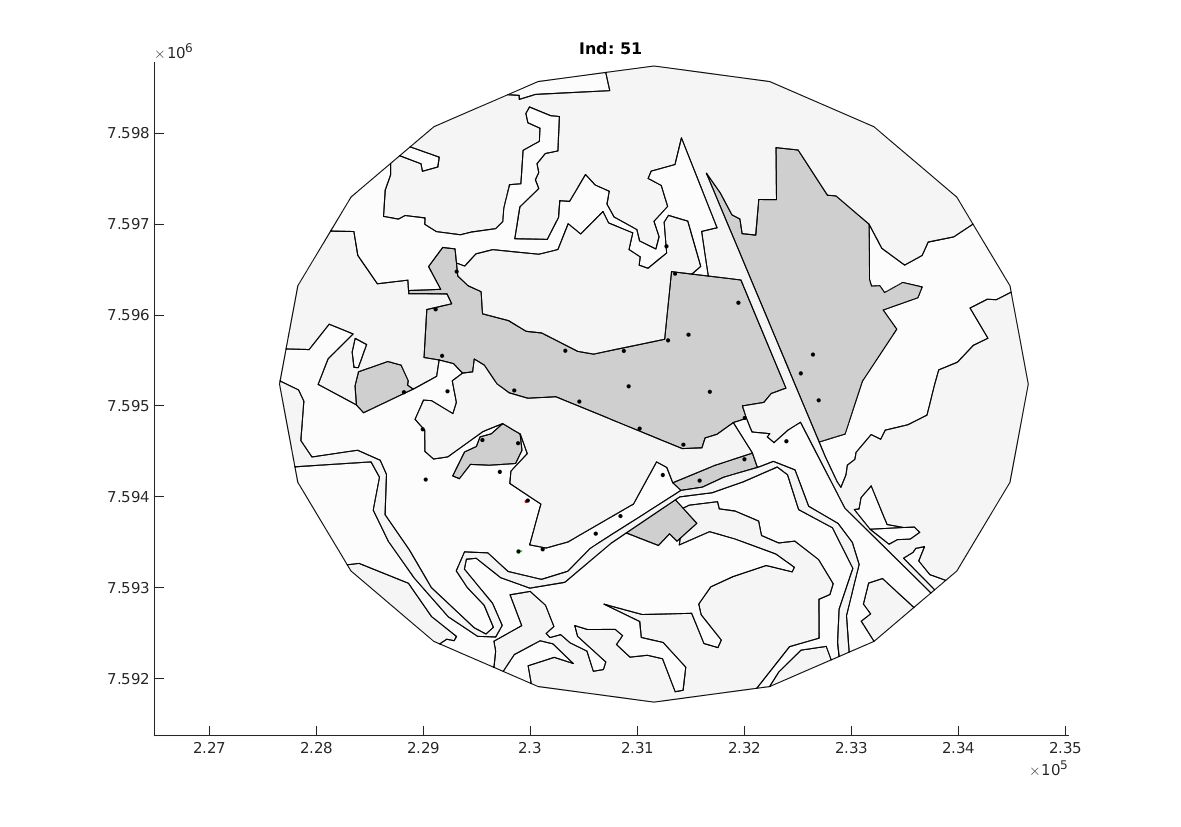

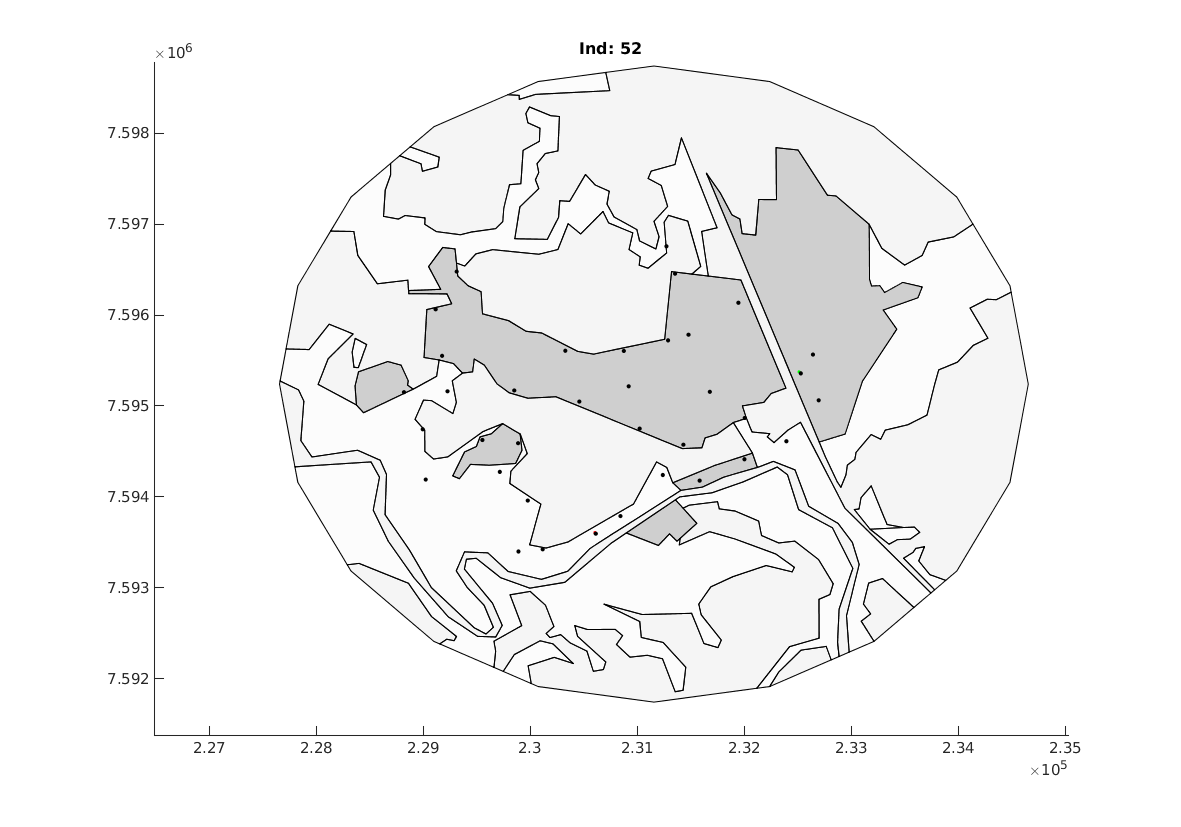

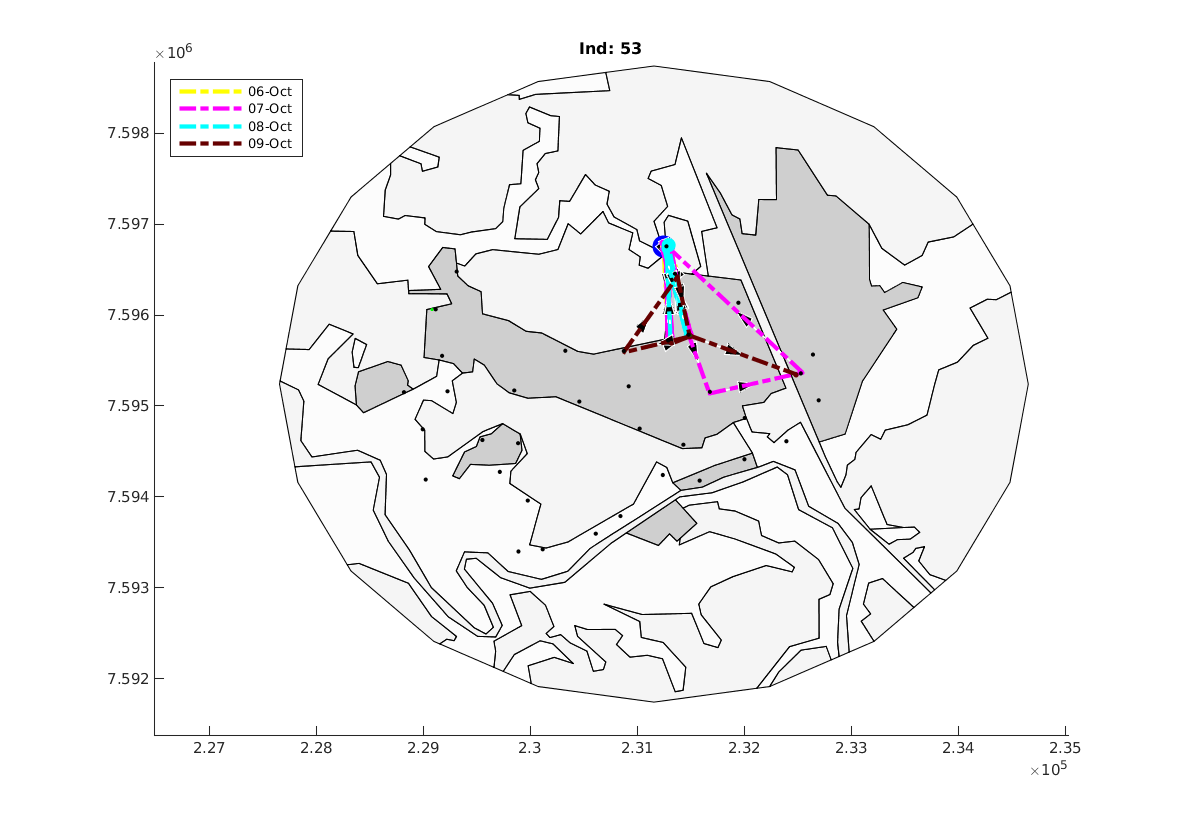

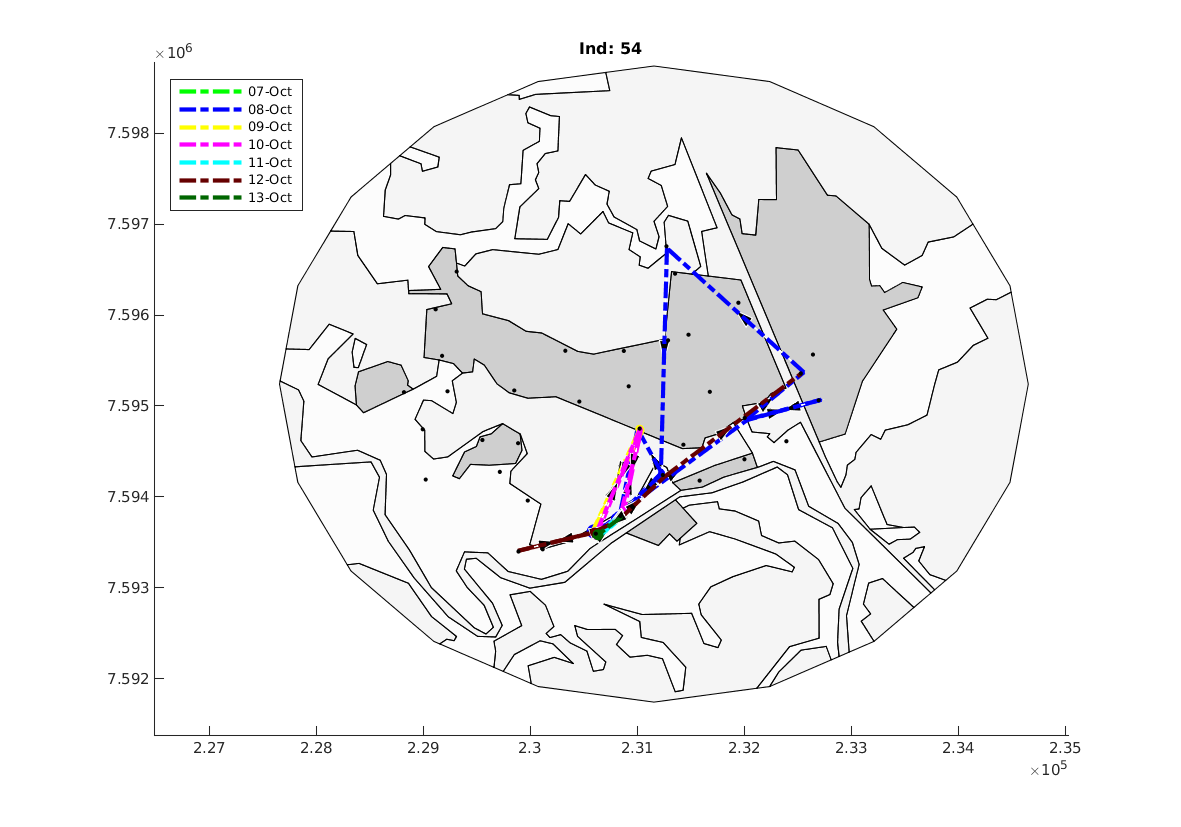

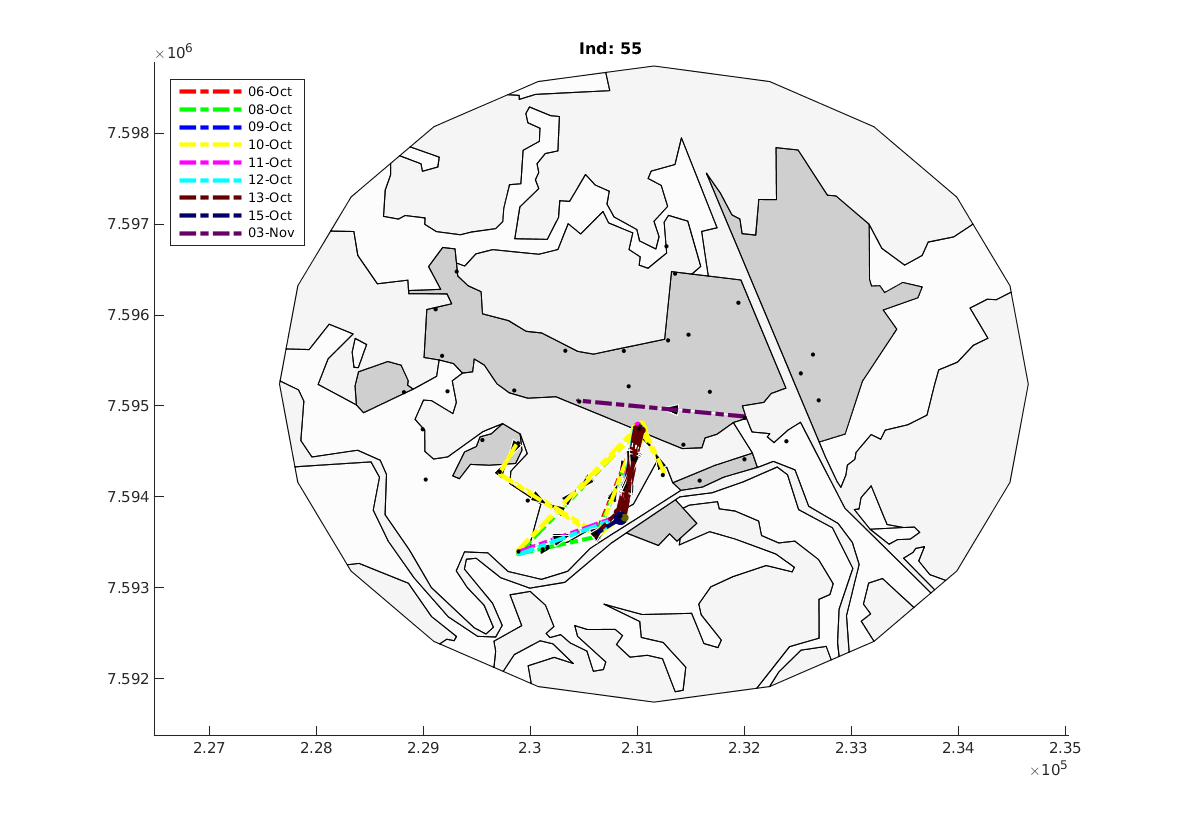

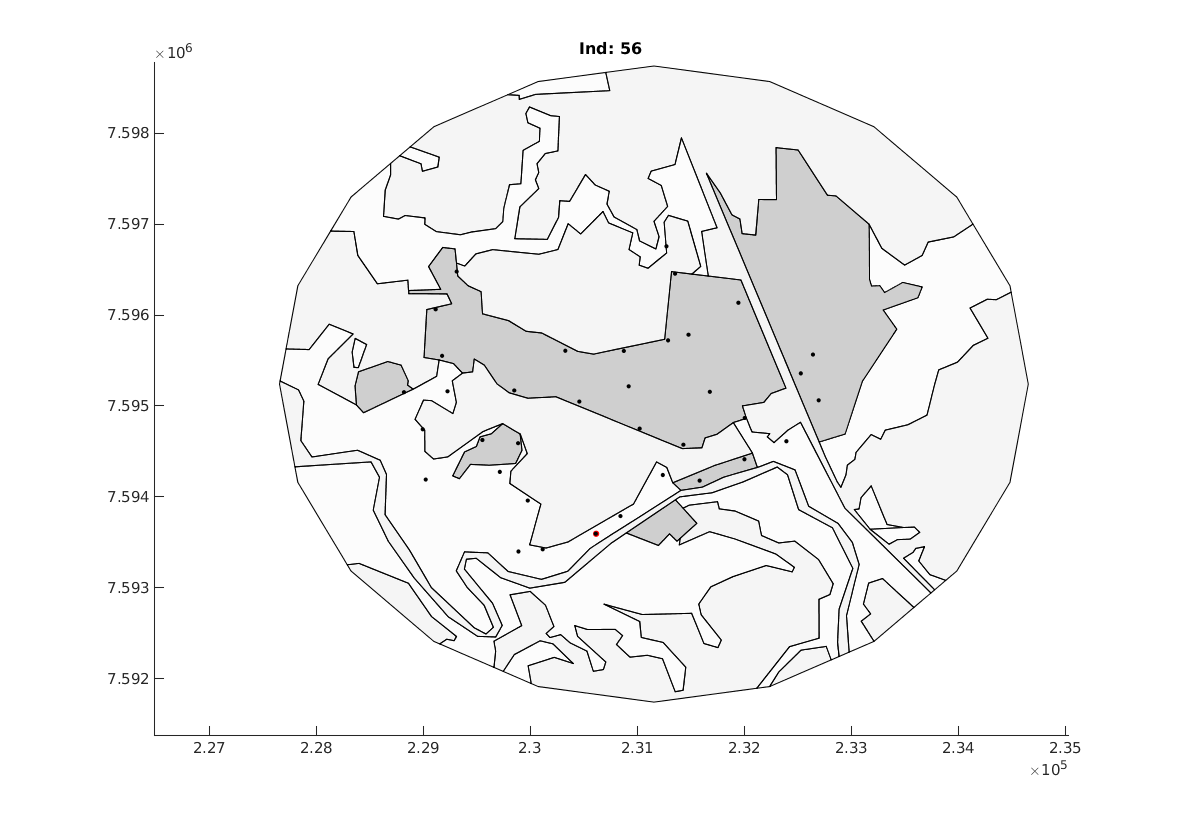

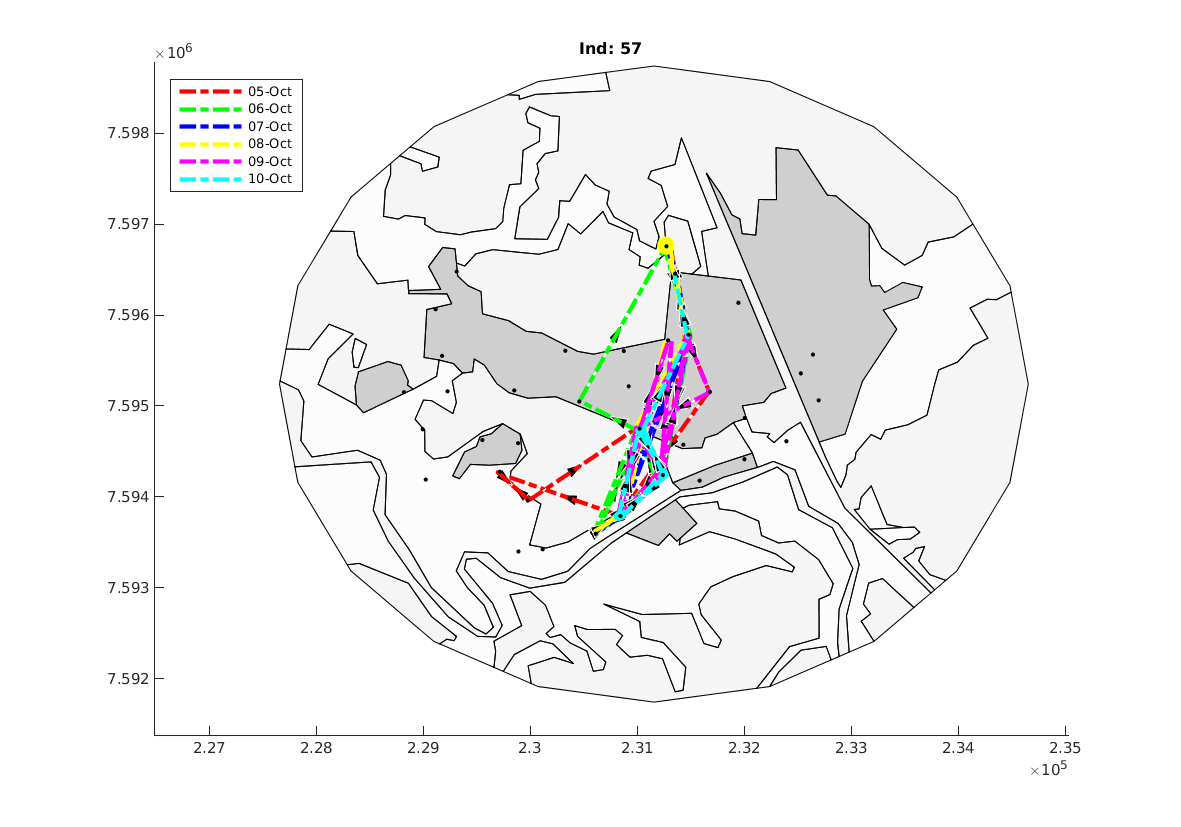

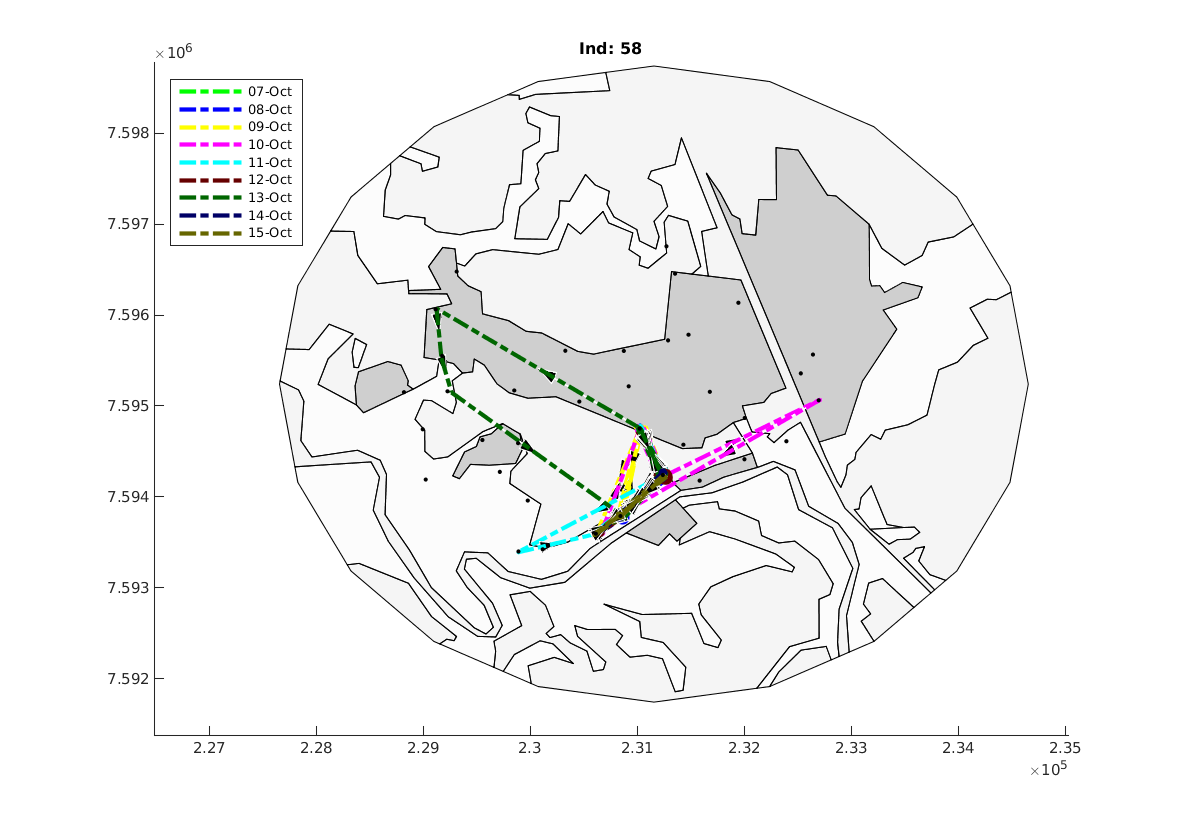

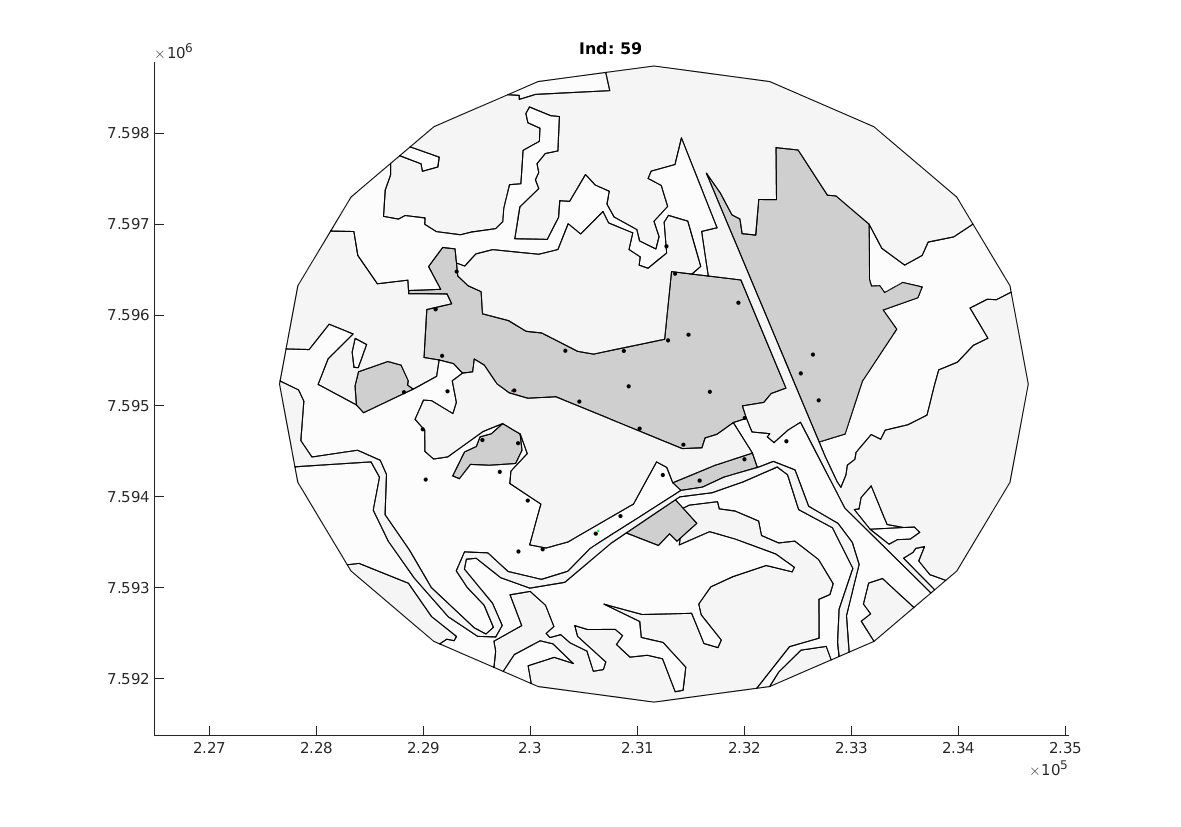

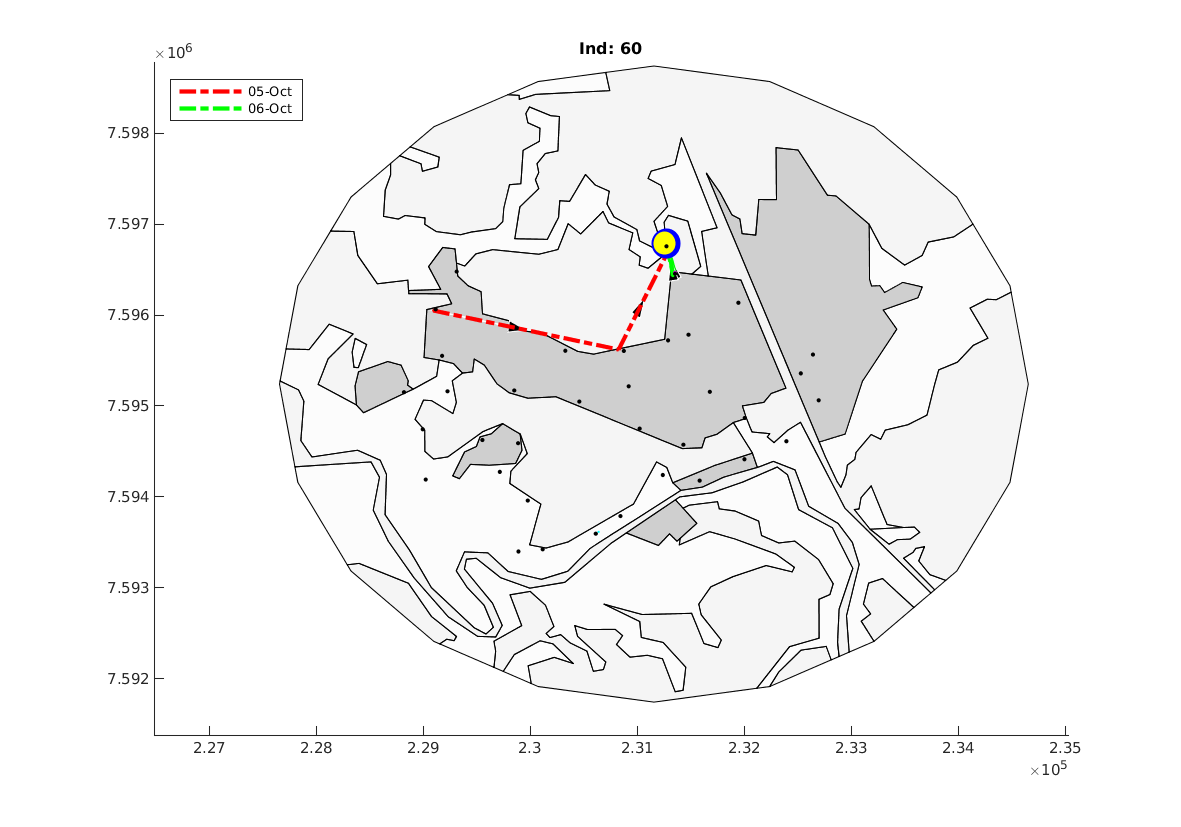

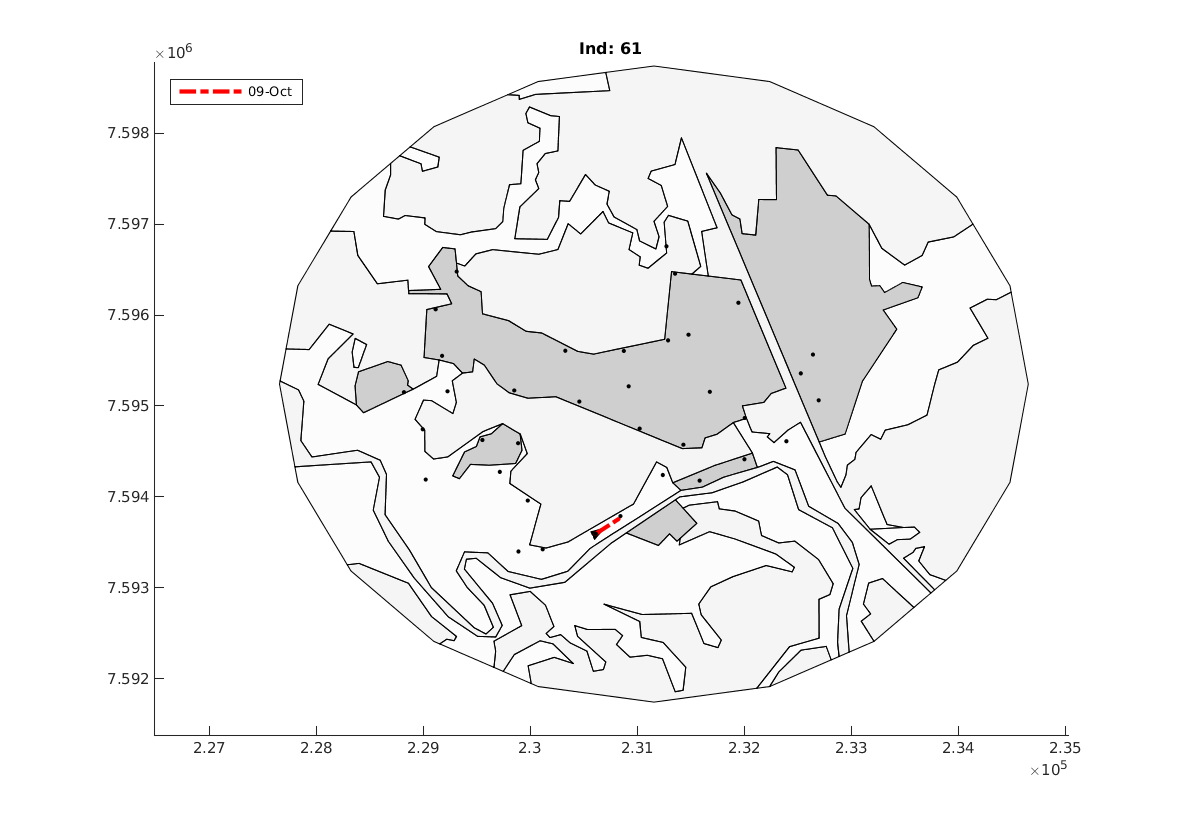

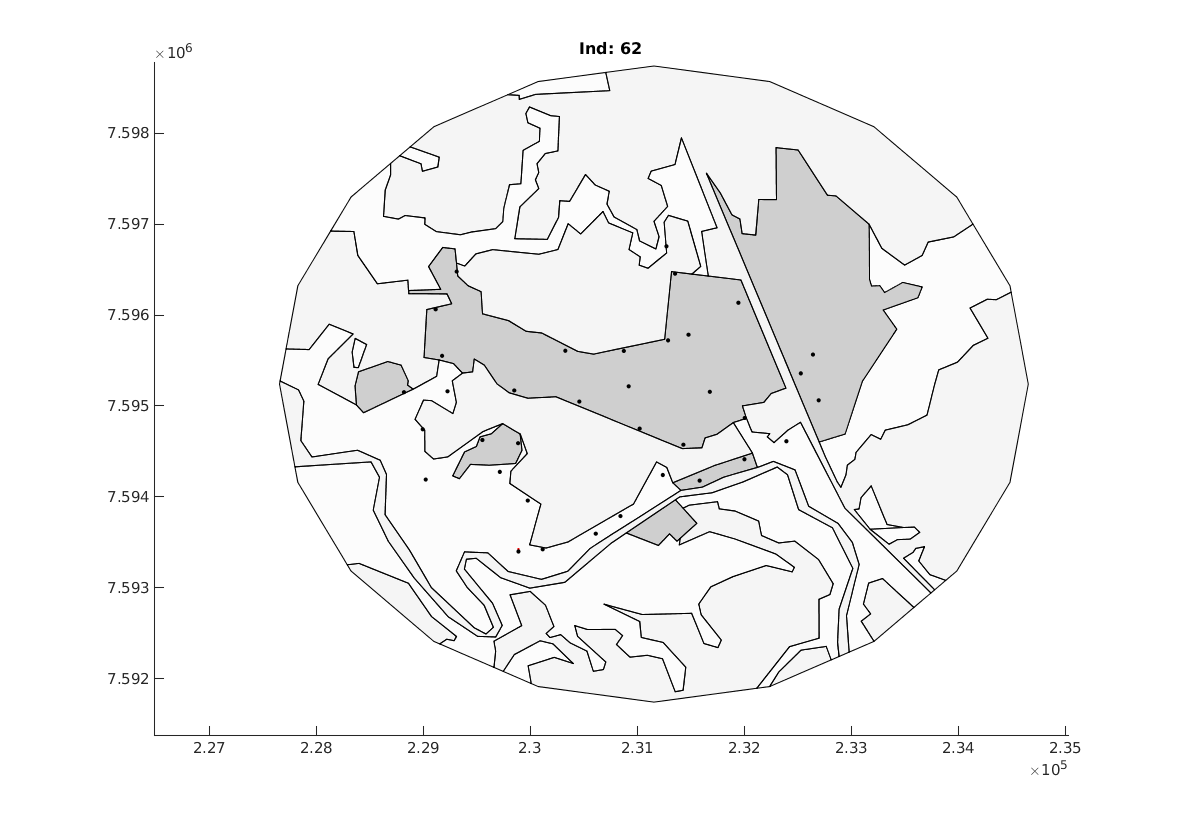

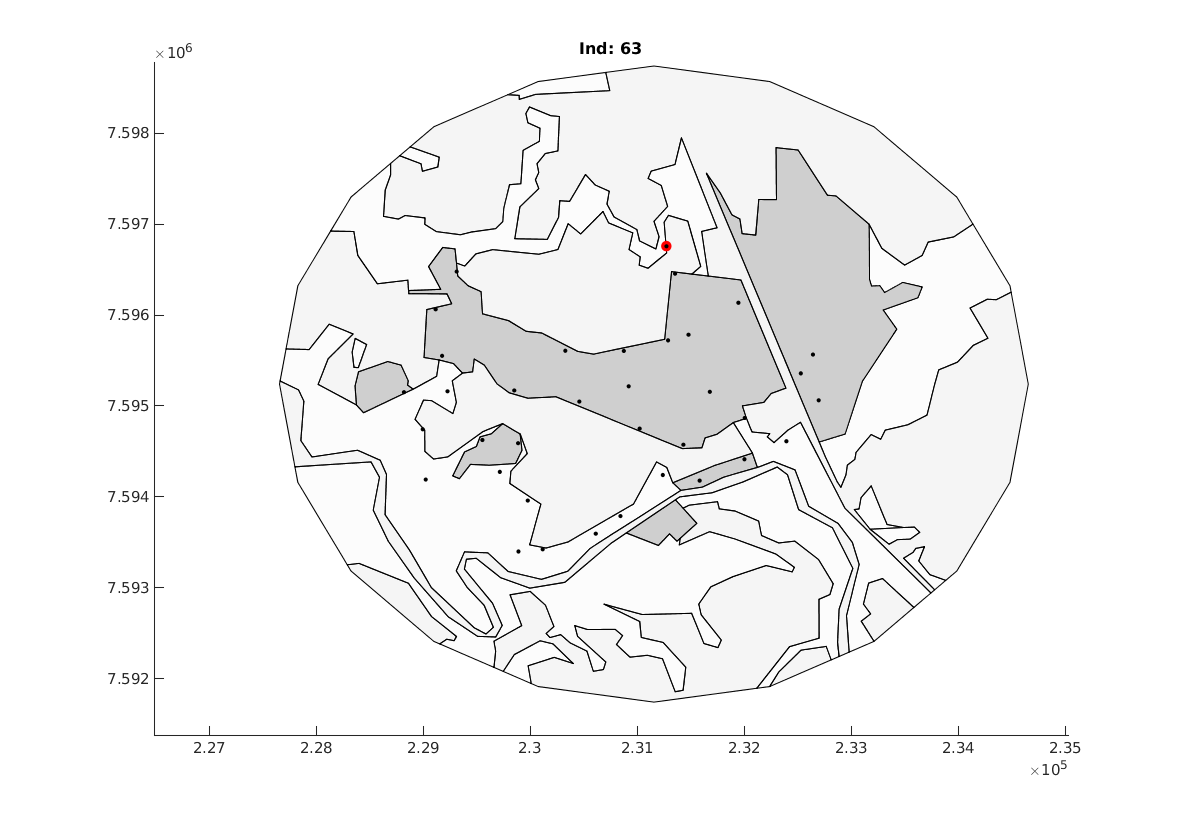

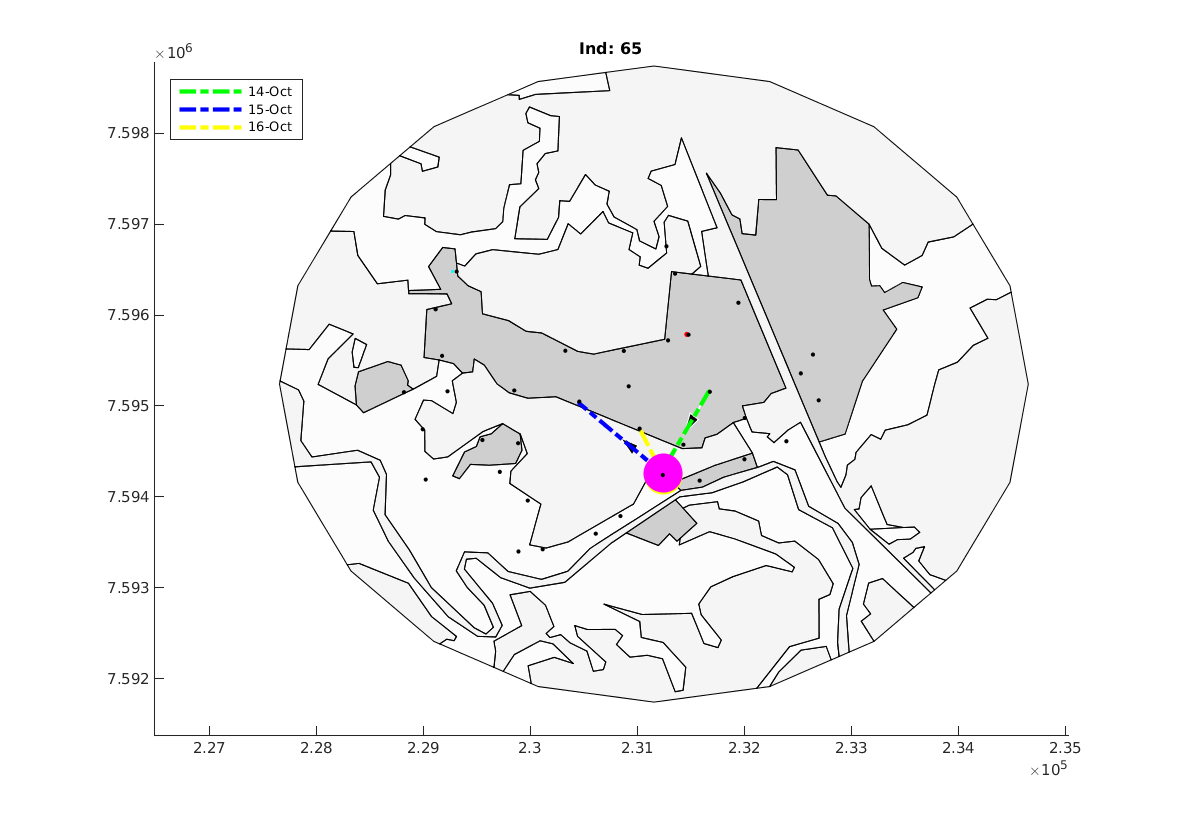

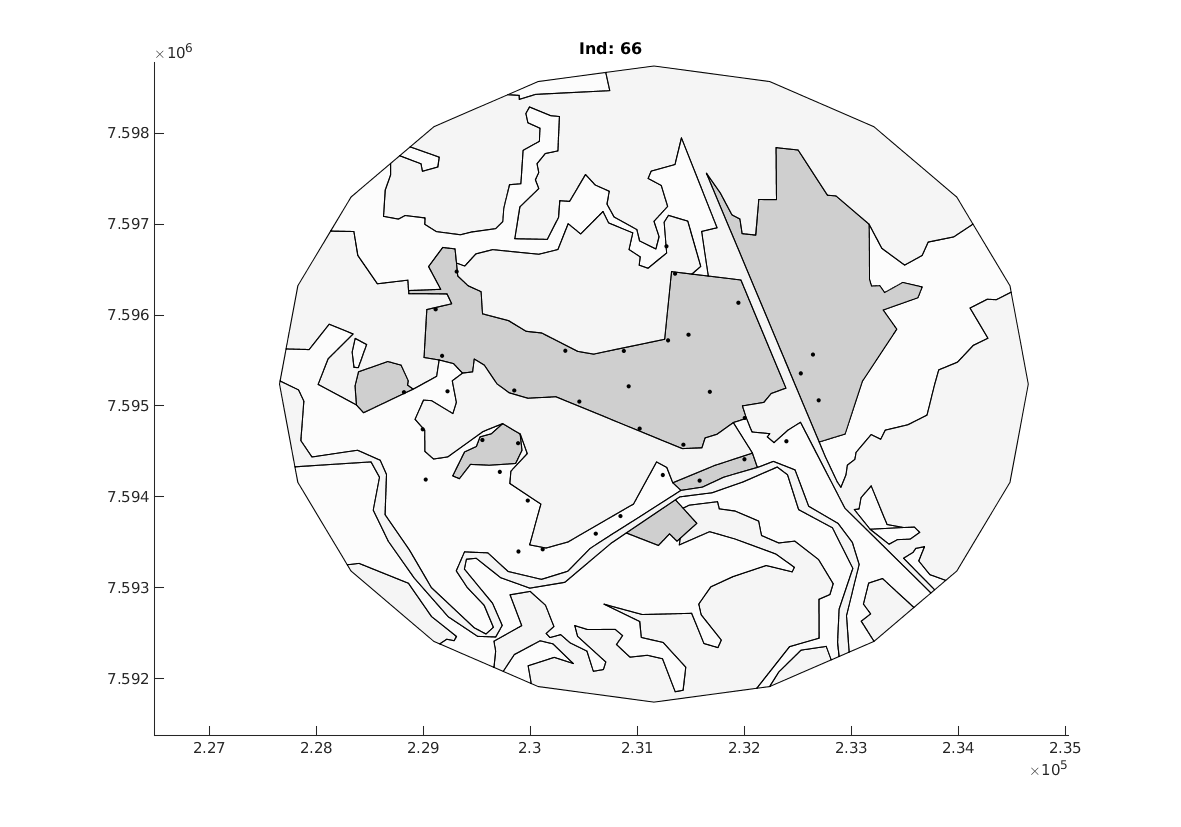

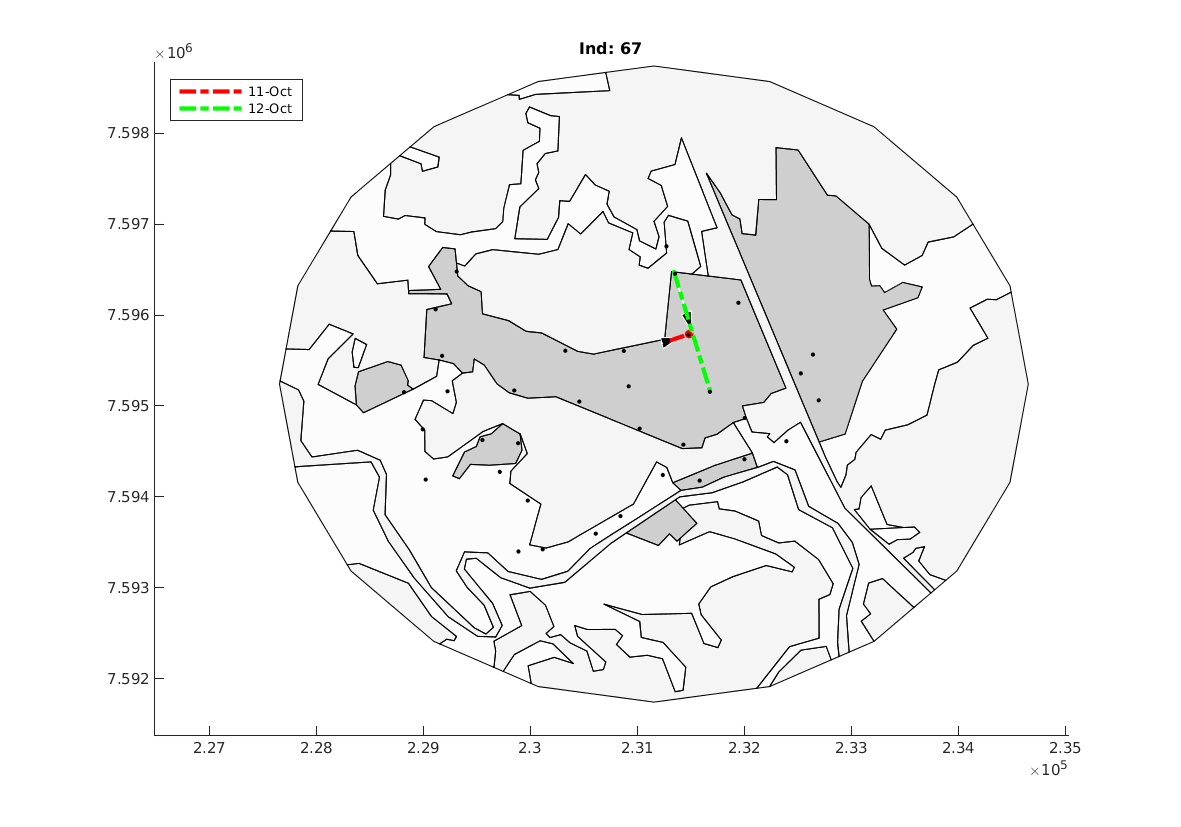

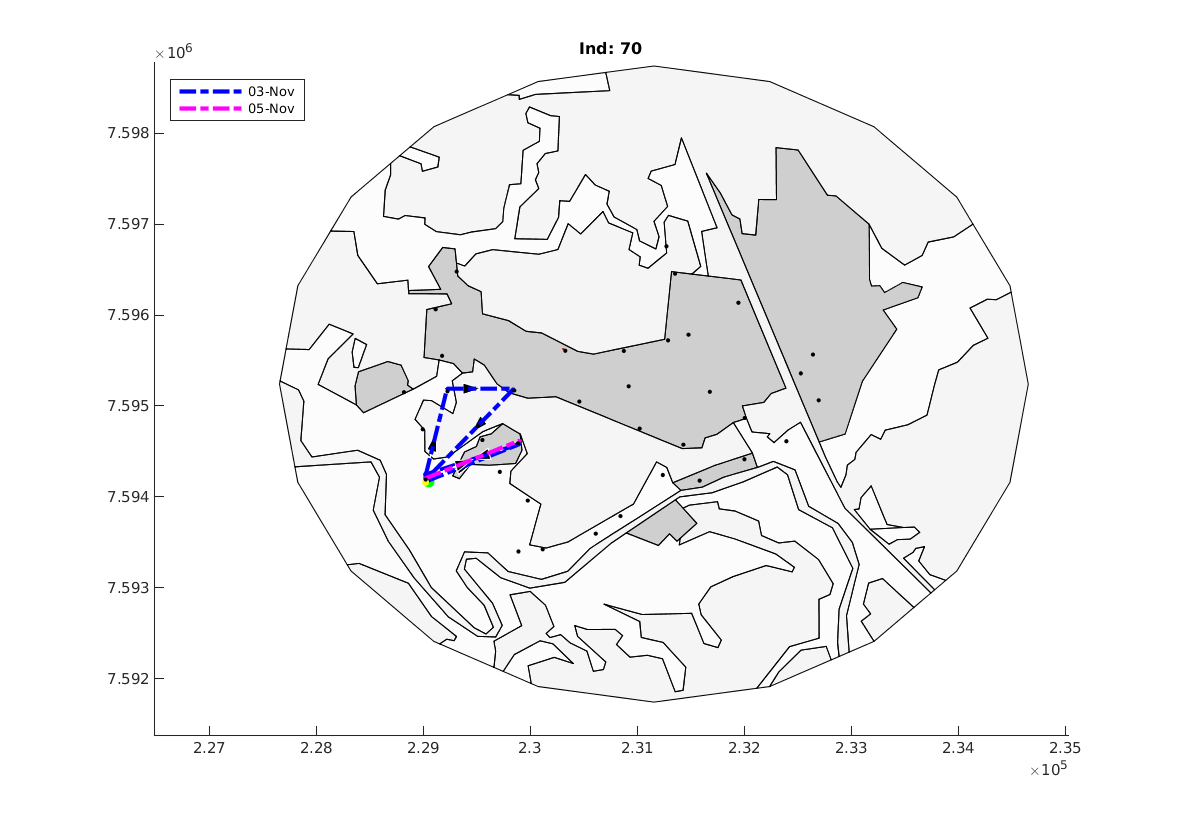

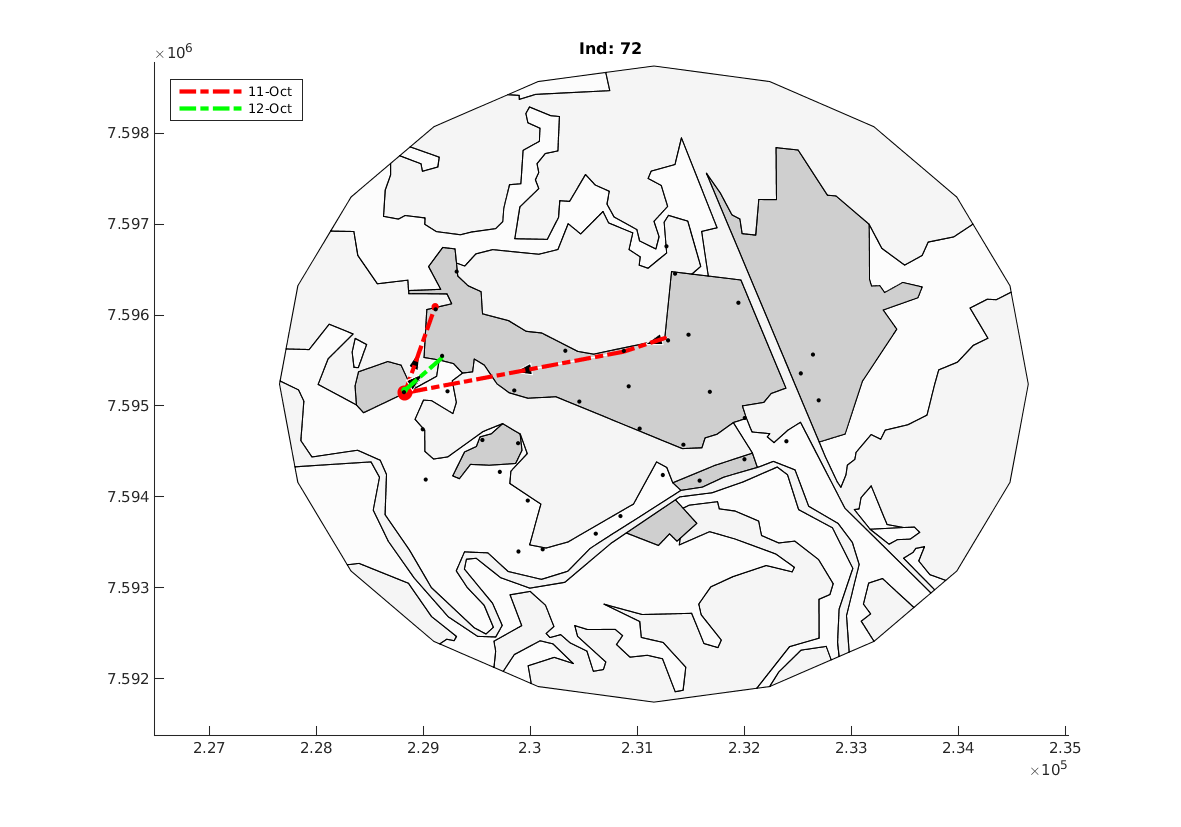

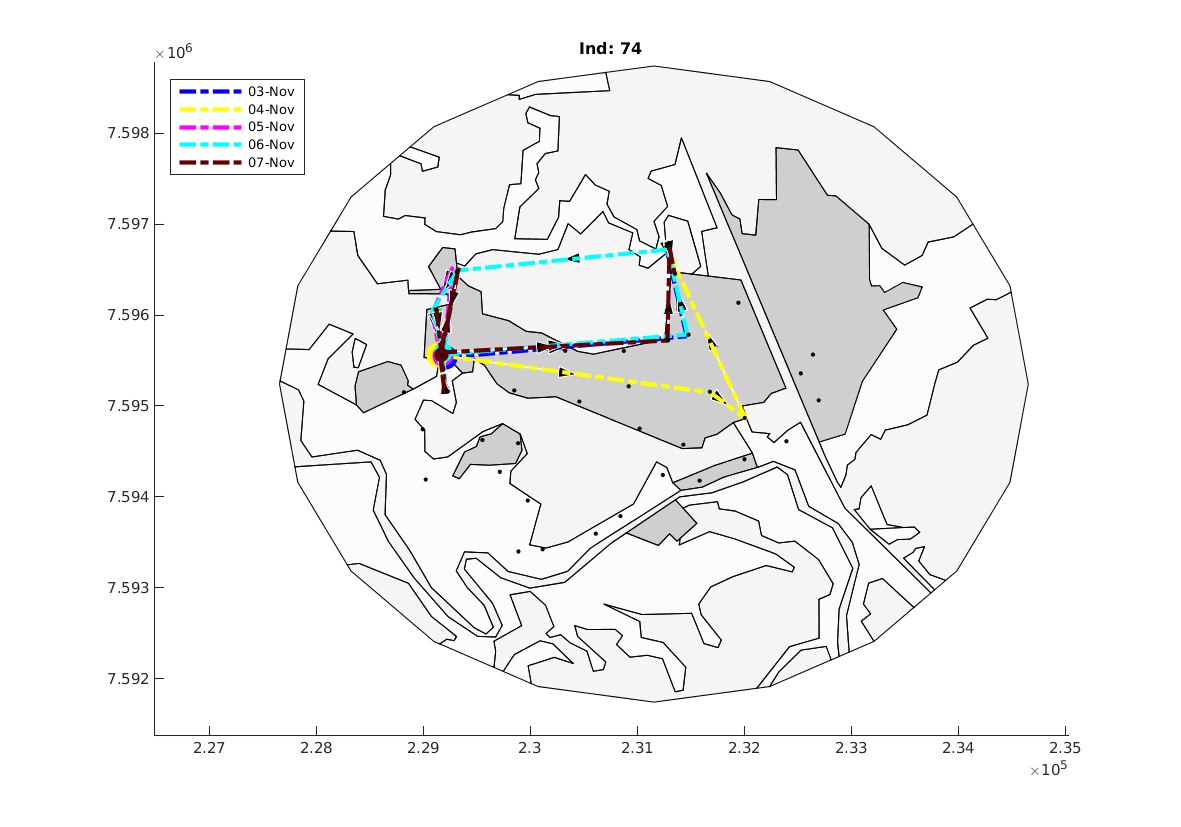

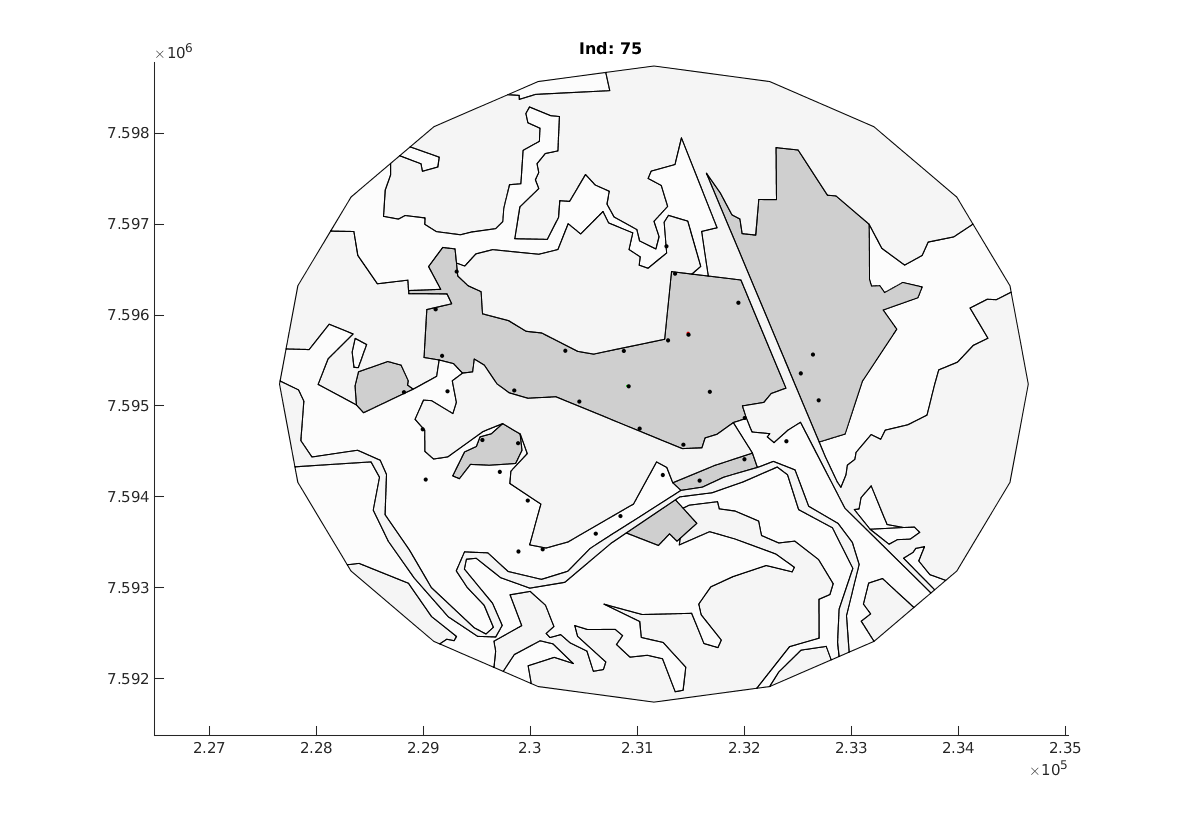

Supplement: Supplementary file 2 — S2. Individuals-movement. [file 40462_2021_266_MOESM2_ESM.docx]
