# Supplementary material for: Movement syndromes of a Neotropical frugivorous bat inhabiting heterogeneous landscapes in Brazil
Source: Mov Ecol. 2021 Jul 7;9:35. doi: 10.1186/s40462-021-00266-6 (PMC8262009; doi:10.1186/s40462-021-00266-6)
Supplement: Supplementary file 3 — S3. Datalogger records summary. [file 40462_2021_266_MOESM3_ESM.docx]

Additional file S3. Movement records and characters summaries of *Sturnira lilium* bats inhabiting a heterogeneous landscape in Brazil. Data were collected by fixed receivers named Dataloggers. ­

| Bat  ID | Number of records | Number of dataloggers | Number of days with records | Days between first and last record | Mark Date (day/month/  year) | Mark month | Sex | Weigh (g) | |  |
| --- | --- | --- | --- | --- | --- | --- | --- | --- | --- | --- |
| 42 | 1 | 1 | 1 | 0 | 09/10/2016 | Oct | M | | 24 |  |
| 43 | 1 | 1 | 1 | 0 | 04/10/2016 | Oct | M | | 23 |  |
| 44 | 45 | 4 | 3 | 2 | 09/10/2016 | Oct | M | | 23 |  |
| 45 | 766 | 10 | 7 | 6 | 09/11/2016 | Nov | M | | 21 |  |
| 46 | 5 | 3 | 2 | 2 | 09/10/2016 | Oct | M | | 22 |  |
| 48 | 102 | 9 | 5 | 4 | 09/10/2016 | Oct | M | | 21 |  |
| 49 | 91 | 6 | 5 | 4 | 30/10/2016 | Oct | M | | 25 |  |
| 50 | 351 | 5 | 4 | 3 | 06/09/2016 | Sep | F | | 21 |  |
| 51 | 1 | 1 | 1 | 0 | 04/10/2016 | Oct | M | | 21 |  |
| 52 | 3 | 2 | 2 | 3 | 09/10/2016 | Oct | M | | 21 |  |
| 53 | 436 | 8 | 7 | 6 | 02/10/2016 | Oct | M | | 24 |  |
| 54 | 353 | 9 | 8 | 7 | 04/10/2016 | Oct | M | | 23 |  |
| 55 | 695 | 8 | 11 | 28 | 04/10/2016 | Oct | F | | 21 |  |
| 56 | 9 | 1 | 1 | 0 | 04/10/2016 | Oct | M | | 23 |  |
| 57 | 451 | 12 | 6 | 5 | 02/10/2016 | Oct | M | | 24 |  |
| 58 | 1655 | 9 | 10 | 9 | 04/10/2016 | Oct | M | | 24 |  |
| 59 | 2 | 2 | 2 | 8 | 04/10/2016 | Oct | M | | 28 |  |
| 60 | 497 | 5 | 6 | 14 | 03/10/2016 | Oct | F | | 20 |  |
| 61 | 6 | 2 | 1 | 0 | 04/10/2016 | Oct | M | | 21 |  |
| 62 | 1 | 1 | 1 | 0 | 04/10/2016 | Oct | M | | 24 |  |
| 63 | 25 | 1 | 1 | 0 | 26/09/2016 | Sep | M | | 24 |  |
| 65 | 1119 | 6 | 6 | 7 | 08/10/2016 | Oct | F | | 22 |  |
| 66 | 0 | 0 | 0 | 0 | 29/10/2016 | Oct | M | | 22 |  |
| 67 | 21 | 4 | 2 | 1 | 08/10/2016 | Oct | M | | 23 |  |
| 70 | 82 | 5 | 5 | 4 | 30/10/2016 | Oct | M | | 22 |  |
| 72 | 70 | 5 | 2 | 1 | 08/10/2016 | Oct | M | | 23 |  |
| 74 | 816 | 11 | 7 | 7 | 26/10/2016 | Oct | M | | 20 |  |
| 75 | 3 | 2 | 2 | 13 | 08/10/2016 | Oct | M | | 27 |  |
