# Supplementary material for: Movement syndromes of a Neotropical frugivorous bat inhabiting heterogeneous landscapes in Brazil
Source: Mov Ecol. 2021 Jul 7;9:35. doi: 10.1186/s40462-021-00266-6 (PMC8262009; doi:10.1186/s40462-021-00266-6)
Supplement: Supplementary file 4 — S4. Clusters definition and PCA output. [file 40462_2021_266_MOESM4_ESM.docx]

Additional file S4. Clusters definition and PCA output

We collected data on movements of 27 Sturnira lilium bats using radio telemetry at a heterogenous landscape in Brazil. Movement variation among individuals could be summarized in four movement syndromes: (1) average type within the population, (2) forest specialists, (3) explorers which prefer *Piper*, and (4) open area specialists which prefer *Solanum* and *Cecropia*.

The optimal number of clusters was defined as follow:

Figure S4-1 – Cluster validation analysis. Silhouette coefficient quantifies the quality of clustering achieved and the dashed line indicates the number of clusters that maximizes the silhouette coefficient.


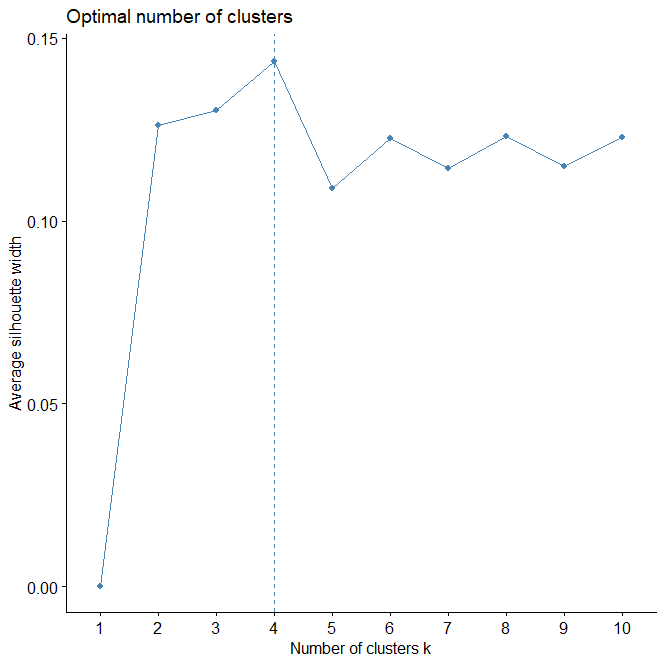


| Table S4-2  PCA Analysis Result  Rotation | (n | X | k) | = | (23 | X | 23): |  |  |  |  |  |  |  |  |  |  |  |  |  |  |  |  |
| --- | --- | --- | --- | --- | --- | --- | --- | --- | --- | --- | --- | --- | --- | --- | --- | --- | --- | --- | --- | --- | --- | --- | --- |
|  | PC1 | PC2 | PC3 | PC4 | PC5 | PC6 | PC7 | PC8 | PC9 | PC10 | PC11 | PC12 | PC13 | PC14 | PC15 | PC16 | PC17 | PC18 | PC19 | PC20 | PC21 | PC22 | PC23 |
| S1 | 0.40 | 0.00 | -0.03 | 0.13 | -0.08 | -0.01 | 0.12 | 0.06 | 0.10 | -0.07 | -0.05 | 0.06 | -0.04 | -0.02 | -0.01 | 0.21 | -0.01 | -0.08 | 0.11 | -0.15 | -0.50 | 0.67 | 0.00 |
| S2 | -0.35 | 0.03 | -0.19 | 0.11 | 0.16 | 0.01 | -0.09 | 0.12 | -0.25 | -0.09 | -0.17 | -0.02 | 0.18 | 0.13 | 0.00 | -0.16 | -0.11 | 0.01 | -0.19 | -0.01 | 0.41 | 0.62 | 0.00 |
| S3 | -0.11 | -0.06 | 0.35 | -0.38 | -0.11 | 0.00 | -0.06 | -0.28 | 0.21 | 0.26 | 0.34 | -0.05 | -0.20 | -0.16 | 0.01 | -0.09 | 0.19 | 0.10 | 0.10 | 0.26 | 0.18 | 0.41 | 0.00 |
| S4 | -0.07 | -0.20 | 0.43 | -0.18 | -0.16 | 0.22 | -0.22 | -0.08 | -0.06 | 0.16 | 0.00 | 0.12 | 0.29 | -0.01 | -0.04 | 0.14 | -0.30 | -0.20 | -0.41 | -0.40 | -0.10 | 0.00 | 0.00 |
| S5 | -0.21 | 0.01 | -0.35 | 0.10 | 0.25 | 0.15 | -0.20 | -0.13 | 0.04 | 0.26 | 0.30 | -0.26 | -0.34 | 0.10 | 0.09 | 0.39 | -0.29 | 0.07 | -0.20 | 0.05 | -0.20 | 0.00 | 0.00 |
| S6 | 0.25 | 0.20 | -0.24 | 0.00 | -0.19 | -0.25 | 0.03 | -0.09 | 0.28 | 0.27 | -0.10 | 0.10 | -0.25 | -0.09 | 0.33 | -0.28 | -0.25 | -0.28 | -0.17 | -0.24 | 0.27 | 0.00 | 0.00 |
| S7 | -0.27 | -0.06 | 0.19 | -0.07 | -0.22 | -0.41 | 0.10 | 0.22 | 0.03 | 0.01 | -0.28 | 0.26 | -0.26 | 0.05 | -0.05 | 0.27 | -0.43 | 0.32 | 0.10 | 0.15 | -0.02 | 0.00 | 0.00 |
| S8 | -0.27 | 0.04 | 0.10 | -0.05 | -0.19 | -0.27 | 0.36 | 0.11 | 0.13 | -0.43 | 0.24 | -0.41 | -0.06 | -0.01 | 0.05 | 0.01 | 0.06 | -0.26 | -0.37 | -0.02 | -0.11 | 0.00 | 0.00 |
| S9 | 0.38 | -0.10 | 0.07 | 0.06 | -0.08 | 0.15 | 0.16 | 0.00 | -0.05 | -0.07 | 0.05 | -0.22 | 0.03 | 0.00 | -0.03 | 0.04 | -0.25 | 0.23 | -0.14 | 0.16 | 0.24 | 0.00 | -0.71 |
| S10 | -0.38 | 0.10 | -0.07 | -0.06 | 0.08 | -0.15 | -0.16 | 0.00 | 0.05 | 0.07 | -0.05 | 0.22 | -0.03 | 0.00 | 0.03 | -0.04 | 0.25 | -0.23 | 0.14 | -0.16 | -0.24 | 0.00 | -0.71 |
| S11 | 0.12 | 0.22 | 0.28 | 0.05 | 0.42 | -0.36 | 0.01 | -0.11 | 0.10 | 0.05 | 0.05 | 0.01 | 0.21 | 0.25 | 0.33 | -0.05 | 0.13 | 0.45 | -0.24 | -0.15 | -0.11 | 0.00 | 0.00 |
| S12 | 0.18 | 0.27 | 0.28 | -0.01 | 0.33 | -0.06 | 0.03 | 0.18 | -0.06 | 0.03 | 0.22 | 0.18 | -0.01 | 0.34 | -0.11 | 0.09 | -0.21 | -0.52 | 0.09 | 0.33 | 0.11 | 0.00 | 0.00 |
| S13 | -0.05 | 0.32 | 0.21 | -0.04 | 0.23 | 0.17 | -0.22 | 0.38 | 0.12 | -0.15 | 0.06 | -0.24 | -0.09 | -0.44 | -0.02 | -0.24 | -0.29 | 0.12 | 0.24 | -0.22 | -0.07 | 0.00 | 0.00 |
| S14 | 0.00 | 0.43 | -0.02 | -0.13 | -0.15 | 0.18 | -0.19 | -0.16 | 0.01 | -0.10 | -0.36 | 0.04 | 0.03 | -0.07 | 0.02 | -0.17 | -0.03 | 0.02 | -0.36 | 0.52 | -0.33 | 0.00 | 0.00 |
| S15 | -0.12 | -0.40 | -0.07 | 0.22 | -0.04 | -0.04 | -0.03 | 0.01 | 0.19 | -0.01 | 0.28 | 0.11 | 0.29 | 0.07 | 0.22 | -0.47 | -0.33 | -0.01 | 0.13 | 0.28 | -0.28 | 0.00 | 0.00 |
| S16 | -0.10 | 0.35 | -0.09 | -0.06 | -0.24 | 0.06 | -0.06 | -0.43 | 0.11 | -0.33 | 0.11 | -0.02 | 0.32 | 0.14 | 0.19 | 0.27 | -0.22 | 0.02 | 0.40 | -0.11 | 0.15 | 0.00 | 0.00 |
| K2 | 0.03 | -0.31 | 0.17 | 0.24 | 0.24 | -0.07 | -0.28 | -0.09 | 0.30 | -0.22 | -0.32 | -0.07 | 0.01 | -0.31 | 0.29 | 0.32 | 0.09 | -0.23 | -0.01 | 0.21 | 0.19 | 0.00 | 0.00 |
| K3 | -0.07 | 0.11 | 0.12 | 0.50 | -0.05 | -0.16 | -0.10 | -0.29 | 0.32 | 0.10 | -0.06 | -0.13 | 0.02 | 0.06 | -0.66 | -0.12 | -0.03 | 0.01 | -0.03 | -0.06 | 0.04 | 0.00 | 0.00 |
| m | -0.07 | -0.05 | -0.17 | -0.22 | 0.41 | 0.20 | 0.44 | -0.21 | 0.31 | -0.20 | 0.01 | 0.42 | 0.01 | -0.22 | -0.22 | 0.01 | -0.14 | 0.06 | -0.16 | -0.05 | 0.04 | 0.00 | 0.00 |
| D1 | -0.06 | 0.09 | 0.20 | 0.45 | -0.18 | 0.29 | -0.08 | 0.00 | -0.08 | -0.29 | 0.26 | 0.44 | -0.41 | 0.05 | 0.18 | -0.05 | 0.14 | 0.11 | -0.15 | -0.08 | 0.09 | 0.00 | 0.00 |
| D2 | -0.13 | 0.27 | 0.02 | 0.33 | -0.10 | -0.03 | 0.30 | 0.11 | -0.13 | 0.39 | 0.17 | 0.11 | 0.36 | -0.50 | 0.10 | 0.24 | 0.06 | -0.01 | -0.05 | 0.15 | 0.03 | 0.00 | 0.00 |
| D3 | -0.18 | 0.05 | 0.13 | 0.06 | -0.07 | 0.47 | 0.28 | 0.26 | 0.47 | 0.25 | -0.26 | -0.13 | 0.03 | 0.37 | 0.18 | 0.03 | 0.11 | 0.01 | 0.08 | -0.01 | 0.05 | 0.00 | 0.00 |
| q | 0.13 | 0.04 | -0.27 | -0.14 | -0.17 | -0.09 | -0.37 | 0.45 | 0.41 | -0.08 | 0.27 | 0.19 | 0.24 | 0.04 | -0.17 | 0.18 | 0.16 | 0.14 | -0.19 | 0.06 | 0.14 | 0.00 | 0.00 |
|  |  |  |  |  |  |  |  |  |  |  |  |  |  |  |  |  |  |  |  |  |  |  |  |
| Importance  of components: |  |  |  |  |  |  |  |  |  |  |  |  |  |  |  |  |  |  |  |  |  |  |  |
|  | PC1 | PC2 | PC3 | PC4 | PC5 | PC6 | PC7 | PC8 | PC9 | PC10 | PC11 | PC12 | PC13 | PC14 | PC15 | PC16 | PC17 | PC18 | PC19 | PC20 | PC21 | PC22 | PC23 |
| Standard deviation | 2.40 | 2.00 | 1.69 | 1.50 | 1.21 | 1.11 | 1.08 | 1.03 | 0.87 | 0.78 | 0.71 | 0.62 | 0.53 | 0.50 | 0.44 | 0.34 | 0.27 | 0.24 | 0.15 | 0.12 | 0.07 | 0.00 | 0.00 |
| Proportion of variance | 0.25 | 0.17 | 0.12 | 0.10 | 0.06 | 0.05 | 0.05 | 0.05 | 0.03 | 0.03 | 0.02 | 0.02 | 0.01 | 0.01 | 0.01 | 0.01 | 0.00 | 0.00 | 0.00 | 0.00 | 0.00 | 0.00 | 0.00 |
| Cumulative proportion | 0.25 | 0.42 | 0.55 | 0.64 | 0.71 | 0.76 | 0.81 | 0.86 | 0.89 | 0.92 | 0.94 | 0.96 | 0.97 | 0.98 | 0.99 | 0.99 | 1.00 | 1.00 | 1.00 | 1.00 | 1.00 | 1.00 | 1.00 |
