# Supplementary material for: Movement syndromes of a Neotropical frugivorous bat inhabiting heterogeneous landscapes in Brazil
Source: Mov Ecol. 2021 Jul 7;9:35. doi: 10.1186/s40462-021-00266-6 (PMC8262009; doi:10.1186/s40462-021-00266-6)
Supplement: Supplementary file 5 — S5. Parameter estimates and convergence diagnostics. [file 40462_2021_266_MOESM5_ESM.docx]

**Supporting Table S6.** Estimated parameters and convergence diagnostics of the MCMC algorithm. The estimated parameters are $\boldsymbol{\Sigma}$, $\zeta_{ap}$, $D_{i}^{1},$ $D_{i}^{2},$ $D_{i}^{3},$ $k_{i}^{2}, k_{i}^{3}, m_{i}$ and $q_{i}$. We show for each parameter the posterior mean, the 0.025 and 0.975 quantiles (p 0.025 and p 0.975), the potential scale reduction factor (Rhat), the upper 95% confidence limit (95% C.I.) and the effective sample size (n.eff).

| ***Sigma*** $\boldsymbol{(}\boldsymbol{\Sigma}_{\boldsymbol{1}\boldsymbol{p}}\mathbf{)}$ |  |  |  |  |  |  |
| --- | --- | --- | --- | --- | --- | --- |
| **Parameter** $\boldsymbol{p}$ | **Mean** | **p 0.025** | **p 0.975** | **Rhat** | **95% C.I.** | **n.eff** |
| 1 | 0.41 | 0.15 | 0.85 | 1.33 | 1.99 | 10500 |
| 2 | 0.08 | -0.15 | 0.33 | 1.19 | 1.53 | 10436 |
| 3 | -0.01 | -0.17 | 0.17 | 1.35 | 2.56 | 10335 |
| 4 | -0.05 | -0.32 | 0.15 | 1.26 | 1.72 | 10500 |
| 5 | -0.11 | -0.52 | 0.15 | 1.04 | 1.08 | 9836 |
| 6 | 0.07 | -0.23 | 0.54 | 1.20 | 1.61 | 10500 |
| 7 | -0.09 | -0.52 | 0.12 | 1.22 | 1.66 | 10816 |
|  |  |  |  |  |  |  |
| ***Sigma*** $\boldsymbol{(}\boldsymbol{\Sigma}_{\boldsymbol{2}\boldsymbol{p}}\mathbf{)}$ |  |  |  |  |  |  |
| **Parameter** $\boldsymbol{p}$ | **Mean** | **p 0.025** | **p 0.975** | **Rhat** | **95% C.I.** | **n.eff** |
| 1 | 0.08 | -0.15 | 0.33 | 1.18 | 1.53 | 10500 |
| 2 | 0.45 | 0.20 | 1.00 | 1.17 | 1.49 | 11130 |
| 3 | 0.01 | -0.20 | 0.16 | 1.19 | 1.54 | 10500 |
| 4 | -0.03 | -0.21 | 0.20 | 1.09 | 1.26 | 10500 |
| 5 | -0.03 | -0.31 | 0.16 | 1.01 | 1.01 | 10500 |
| 6 | 0.08 | -0.30 | 0.49 | 1.14 | 1.40 | 10500 |
| 7 | -0.05 | -0.55 | 0.26 | 1.11 | 1.28 | 11035 |
|  |  |  |  |  |  |  |
| ***Sigma*** $\boldsymbol{(}\boldsymbol{\Sigma}_{\boldsymbol{3}\boldsymbol{p}}\mathbf{)}$ |  |  |  |  |  |  |
| **Parameter** $\boldsymbol{p}$ | **Mean** | **p 0.025** | **p 0.975** | **Rhat** | **95% C.I.** | **n.eff** |
| 1 | -0.01 | -0.17 | 0.17 | 1.36 | 2.66 | 10500 |
| 2 | 0.01 | -0.20 | 0.16 | 1.20 | 1.57 | 10500 |
| 3 | 0.22 | 0.10 | 0.42 | 1.49 | 2.48 | 10315 |
| 4 | -0.05 | -0.22 | 0.09 | 1.12 | 1.26 | 10760 |
| 5 | 0.01 | -0.10 | 0.15 | 1.05 | 1.10 | 10971 |
| 6 | 0.00 | -0.20 | 0.17 | 1.19 | 1.54 | 10320 |
| 7 | -0.01 | -0.17 | 0.14 | 1.07 | 1.19 | 10512 |
|  |  |  |  |  |  |  |
| ***Sigma*** $\boldsymbol{(}\boldsymbol{\Sigma}_{\boldsymbol{4}\boldsymbol{p}}\mathbf{)}$ |  |  |  |  |  |  |
| **Parameter** $\boldsymbol{p}$ | **Mean** | **p 0.025** | **p 0.975** | **Rhat** | **95% C.I.** | **n.eff** |
| 1 | -0.05 | -0.32 | 0.15 | 1.28 | 1.79 | 10677 |
| 2 | -0.03 | -0.21 | 0.20 | 1.09 | 1.27 | 10871 |
| 3 | -0.05 | -0.22 | 0.09 | 1.11 | 1.23 | 10487 |
| 4 | 0.35 | 0.19 | 0.67 | 1.12 | 1.25 | 10289 |
| 5 | 0.05 | -0.13 | 0.29 | 1.11 | 1.33 | 10950 |
| 6 | -0.02 | -0.32 | 0.23 | 1.09 | 1.27 | 11365 |
| 7 | 0.10 | -0.19 | 0.55 | 1.13 | 1.36 | 10500 |
|  |  |  |  |  |  |  |
| ***Sigma*** $\boldsymbol{(}\boldsymbol{\Sigma}_{\boldsymbol{5}\boldsymbol{p}}\mathbf{)}$ |  |  |  |  |  |  |
| **Parameter** $\boldsymbol{p}$ | **Mean** | **p 0.025** | **p 0.975** | **Rhat** | **95% C.I.** | **n.eff** |
| 1 | -0.11 | -0.52 | 0.15 | 1.05 | 1.11 | 10500 |
| 2 | -0.03 | -0.31 | 0.16 | 1.01 | 1.01 | 10209 |
| 3 | 0.01 | -0.10 | 0.15 | 1.05 | 1.10 | 10500 |
| 4 | 0.05 | -0.13 | 0.29 | 1.10 | 1.31 | 10248 |
| 5 | 0.36 | 0.09 | 0.85 | 1.11 | 1.33 | 10285 |
| 6 | -0.06 | -0.32 | 0.11 | 1.43 | 2.23 | 10466 |
| 7 | 0.00 | -0.24 | 0.22 | 1.13 | 1.40 | 10500 |
|  |  |  |  |  |  |  |
| ***Sigma*** $\boldsymbol{(}\boldsymbol{\Sigma}_{\boldsymbol{6}\boldsymbol{p}}\mathbf{)}$ |  |  |  |  |  |  |
| **Parameter** $\boldsymbol{p}$ | **Mean** | **p 0.025** | **p 0.975** | **Rhat** | **95% C.I.** | **n.eff** |
| 1 | 0.07 | -0.23 | 0.54 | 1.19 | 1.57 | 10599 |
| 2 | 0.08 | -0.30 | 0.49 | 1.14 | 1.41 | 10500 |
| 3 | 0.00 | -0.20 | 0.17 | 1.18 | 1.52 | 11197 |
| 4 | -0.02 | -0.32 | 0.23 | 1.09 | 1.25 | 10805 |
| 5 | -0.06 | -0.32 | 0.11 | 1.45 | 2.25 | 10325 |
| 6 | 0.44 | 0.18 | 0.97 | 1.22 | 1.69 | 10500 |
| 7 | -0.13 | -0.89 | 0.20 | 1.32 | 2.60 | 10317 |
|  |  |  |  |  |  |  |
| ***Sigma*** $\boldsymbol{(}\boldsymbol{\Sigma}_{\boldsymbol{7}\boldsymbol{p}}\mathbf{)}$ |  |  |  |  |  |  |
| **Parameter** $\boldsymbol{p}$ | **Mean** | **p 0.025** | **p 0.975** | **Rhat** | **95% C.I.** | **n.eff** |
| 1 | -0.09 | -0.52 | 0.12 | 1.24 | 1.71 | 10500 |
| 2 | -0.05 | -0.55 | 0.26 | 1.12 | 1.32 | 10994 |
| 3 | -0.01 | -0.17 | 0.14 | 1.07 | 1.19 | 10673 |
| 4 | 0.10 | -0.19 | 0.55 | 1.15 | 1.44 | 9434 |
| 5 | 0.00 | -0.24 | 0.22 | 1.12 | 1.36 | 10813 |
| 6 | -0.13 | -0.89 | 0.20 | 1.32 | 2.65 | 10500 |
| 7 | 0.55 | 0.17 | 1.62 | 1.34 | 2.69 | 10696 |
|  |  |  |  |  |  |  |
| ***Zeta - intercept*** $\boldsymbol{( \zeta}_{\boldsymbol{1p}}\boldsymbol{)}$ | | | |  |  |  |
| **Parameter** $\boldsymbol{p}$ | **Mean** | **p 0.025** | **p 0.975** | **Rhat** | **95% C.I.** | **n.eff** |
| 1 | -0.49 | -13.61 | 13.95 | 1.09 | 1.25 | 10808 |
| 2 | 0.49 | -9.91 | 13.31 | 1.10 | 1.30 | 10500 |
| 3 | -0.45 | -6.96 | 6.29 | 1.10 | 1.31 | 10500 |
| 4 | 10.95 | -2.50 | 24.59 | 1.07 | 1.20 | 10500 |
| 5 | 9.84 | -2.31 | 20.89 | 1.05 | 1.16 | 10502 |
| 6 | 9.19 | -2.20 | 20.02 | 1.08 | 1.26 | 10607 |
| 7 | 2.32 | -6.39 | 12.87 | 1.00 | 1.00 | 10500 |
|  |  |  |  |  |  |  |
| ***Zeta – effect of sex*** $\boldsymbol{( \zeta}_{\boldsymbol{2}\boldsymbol{p}}\boldsymbol{)}$ | | | |  |  |  |
| **Parameter** $\boldsymbol{p}$ | **Mean** | **p 0.025** | **p 0.975** | **Rhat** | **95% C.I.** | **n.eff** |
| 1 | -0.37 | -1.61 | 0.81 | 1.04 | 1.11 | 10305 |
| 2 | 0.25 | -0.75 | 1.20 | 1.08 | 1.22 | 10691 |
| 3 | 0.39 | -0.48 | 1.27 | 1.05 | 1.14 | 10829 |
| 4 | 0.04 | -0.79 | 0.95 | 1.07 | 1.21 | 10755 |
| 5 | 0.01 | -0.88 | 0.77 | 1.01 | 1.03 | 10308 |
| 6 | 0.20 | -0.93 | 1.85 | 1.08 | 1.21 | 10500 |
| 7 | 0.03 | -1.11 | 0.91 | 1.05 | 1.14 | 10843 |
|  |  |  |  |  |  |  |
| ***Zeta – effect of weight*** $\boldsymbol{( \zeta}_{\boldsymbol{3}\boldsymbol{p}}\boldsymbol{)}$ | | | |  |  |  |
| **Parameter** $\boldsymbol{p}$ | **Mean** | **p 0.025** | **p 0.975** | **Rhat** | **95% C.I.** | **n.eff** |
| 1 | 0.26 | -4.51 | 4.72 | 1.07 | 1.20 | 9717 |
| 2 | 1.19 | -3.00 | 4.48 | 1.09 | 1.27 | 10747 |
| 3 | -0.58 | -2.78 | 1.51 | 1.08 | 1.26 | 10537 |
| 4 | 0.53 | -4.07 | 4.94 | 1.08 | 1.21 | 10500 |
| 5 | 0.92 | -2.69 | 4.94 | 1.07 | 1.21 | 10696 |
| 6 | 0.97 | -2.35 | 4.38 | 1.10 | 1.30 | 10500 |
| 7 | -0.12 | -3.48 | 2.75 | 1.00 | 1.01 | 11619 |
|  |  |  |  |  |  |  |
| ***Relative preferences to matrix*** $\boldsymbol{(}\boldsymbol{k}_{\boldsymbol{i}}^{\boldsymbol{2}}\boldsymbol{)}$ | | | |  |  |  |
| **Individual** $\boldsymbol{i}$ | **Mean** | **p 0.025** | **p 0.975** | **Rhat** | **95% C.I.** | **n.eff** |
| 1 | 0.02 | -0.18 | 0.36 | 1.46 | 2.87 | 9780 |
| 2 | -0.01 | -0.43 | 0.63 | 1.62 | 2.72 | 10674 |
| 3 | 0.01 | -1.10 | 0.98 | 1.11 | 1.30 | 10500 |
| 4 | 0.03 | -0.31 | 0.32 | 1.88 | 4.31 | 11068 |
| 5 | 0.25 | -0.11 | 0.55 | 1.52 | 2.30 | 10951 |
| 6 | -0.05 | -1.25 | 1.44 | 1.10 | 1.29 | 10500 |
| 7 | -0.34 | -1.31 | 0.27 | 1.33 | 2.15 | 11164 |
| 8 | -0.08 | -0.50 | 0.15 | 1.68 | 4.17 | 10317 |
| 9 | -0.04 | -0.21 | 0.13 | 1.55 | 2.91 | 10686 |
| 10 | 0.05 | -1.12 | 1.03 | 1.29 | 1.99 | 10943 |
| 11 | -0.22 | -0.53 | 0.20 | 1.22 | 1.62 | 10500 |
| 12 | 0.02 | -0.68 | 0.30 | 1.28 | 2.14 | 10500 |
| 13 | 0.42 | -0.03 | 1.44 | 1.22 | 1.70 | 10811 |
| 14 | -0.38 | -0.87 | 0.06 | 1.31 | 2.87 | 10697 |
| 15 | 0.57 | 0.05 | 1.53 | 2.04 | 7.18 | 10543 |
| 16 | -0.61 | -1.49 | 0.05 | 1.28 | 2.03 | 9745 |
| 17 | 0.03 | -0.68 | 0.52 | 1.18 | 1.42 | 10500 |
| 18 | 0.44 | 0.04 | 1.10 | 1.84 | 3.24 | 10500 |
| 19 | 0.27 | -0.33 | 1.13 | 1.45 | 2.56 | 10299 |
| 20 | 0.28 | 0.04 | 0.57 | 1.24 | 1.75 | 10398 |
| 21 | -0.06 | -0.99 | 0.70 | 1.14 | 1.31 | 10500 |
| 22 | 0.40 | -0.29 | 1.88 | 1.21 | 1.77 | 10291 |
| 23 | 0.10 | -0.68 | 0.94 | 1.21 | 1.59 | 10681 |
| 24 | 0.16 | -0.49 | 0.58 | 1.35 | 1.95 | 10500 |
| 25 | 0.01 | -0.50 | 0.55 | 1.13 | 1.29 | 10925 |
| 26 | -1.01 | -2.07 | 0.16 | 1.29 | 2.00 | 10509 |
| 27 | 0.04 | -0.71 | 0.78 | 1.10 | 1.30 | 10500 |
|  |  |  |  |  |  |  |
| ***Relative preferences to open areas*** $\boldsymbol{(}\boldsymbol{k}_{\boldsymbol{i}}^{\boldsymbol{3}}\boldsymbol{)}$ | | | |  |  |  |
| **Individual** $\boldsymbol{i}$ | **Mean** | **p 0.025** | **p 0.975** | **Rhat** | **95% C.I.** | **n.eff** |
| 1 | 4.59 | 3.05 | 5.45 | 1.10 | 1.22 | 10500 |
| 2 | 4.50 | 3.75 | 5.01 | 1.32 | 2.19 | 10889 |
| 3 | 4.84 | 4.08 | 5.33 | 1.21 | 1.63 | 10500 |
| 4 | 4.54 | 3.71 | 4.98 | 1.21 | 1.69 | 9995 |
| 5 | 4.98 | 4.96 | 5.00 | 1.52 | 2.81 | 10927 |
| 6 | 4.00 | 3.18 | 5.02 | 1.84 | 6.76 | 10913 |
| 7 | 4.27 | 2.92 | 5.06 | 1.18 | 1.64 | 10500 |
| 8 | 4.27 | 3.09 | 4.97 | 1.25 | 1.83 | 10500 |
| 9 | 4.11 | 3.18 | 4.62 | 1.98 | 3.91 | 10772 |
| 10 | 4.38 | 3.99 | 4.93 | 1.12 | 1.19 | 10333 |
| 11 | 4.61 | 3.48 | 5.15 | 1.18 | 1.44 | 10701 |
| 12 | 4.69 | 3.94 | 5.00 | 1.22 | 1.79 | 11182 |
| 13 | 4.25 | 3.51 | 5.00 | 1.11 | 1.31 | 10697 |
| 14 | 4.38 | 3.36 | 5.04 | 1.07 | 1.22 | 10500 |
| 15 | 4.64 | 3.75 | 5.42 | 1.36 | 2.13 | 10714 |
| 16 | 5.02 | 4.88 | 5.24 | 1.17 | 1.48 | 11468 |
| 17 | 4.61 | 3.96 | 5.06 | 1.08 | 1.20 | 10585 |
| 18 | 4.20 | 3.45 | 4.99 | 1.10 | 1.29 | 11551 |
| 19 | 4.42 | 3.51 | 5.02 | 1.16 | 1.49 | 10500 |
| 20 | 4.41 | 3.99 | 5.04 | 1.29 | 2.24 | 10344 |
| 21 | 4.66 | 3.92 | 5.10 | 1.78 | 3.58 | 10270 |
| 22 | 3.78 | 2.57 | 5.00 | 2.32 | 9.96 | 10104 |
| 23 | 4.22 | 3.30 | 5.07 | 1.02 | 1.03 | 10500 |
| 24 | 4.37 | 3.84 | 4.93 | 1.30 | 1.89 | 10500 |
| 25 | 4.21 | 3.23 | 4.92 | 1.39 | 2.38 | 11019 |
| 26 | 4.03 | 3.15 | 5.06 | 1.04 | 1.06 | 10742 |
| 27 | 4.57 | 3.41 | 5.50 | 1.65 | 3.03 | 10750 |
|  |  |  |  |  |  |  |
| ***Mortality*** $\boldsymbol{(}\boldsymbol{m}_{\boldsymbol{i}}\boldsymbol{)}$ | |  |  |  |  |  |
| **Individual** $\boldsymbol{i}$ | **Mean** | **p 0.025** | **p 0.975** | **Rhat** | **95% C.I.** | **n.eff** |
| 1 | -1.96 | -2.05 | -1.66 | 4.46 | 9.44 | 10500 |
| 2 | -1.81 | -1.92 | -1.67 | 1.39 | 2.37 | 10500 |
| 3 | -1.86 | -2.43 | -1.51 | 1.12 | 1.34 | 10096 |
| 4 | -1.80 | -2.22 | -1.28 | 1.06 | 1.10 | 10932 |
| 5 | -2.10 | -2.37 | -1.96 | 1.36 | 3.09 | 10500 |
| 6 | -1.89 | -2.03 | -1.56 | 1.09 | 1.23 | 10500 |
| 7 | -1.73 | -2.38 | -0.53 | 1.18 | 1.57 | 10500 |
| 8 | -1.91 | -2.29 | -1.37 | 1.37 | 2.13 | 10500 |
| 9 | -2.01 | -2.57 | -1.75 | 1.13 | 1.21 | 10500 |
| 10 | -1.75 | -2.23 | -1.33 | 1.32 | 1.88 | 9905 |
| 11 | -2.30 | -2.57 | -2.06 | 2.70 | 4.88 | 10500 |
| 12 | -2.01 | -2.26 | -1.71 | 1.32 | 1.94 | 10092 |
| 13 | -2.48 | -3.52 | -1.95 | 1.31 | 2.24 | 10735 |
| 14 | -1.56 | -2.10 | -0.66 | 1.18 | 1.55 | 10200 |
| 15 | -1.62 | -1.99 | -1.02 | 1.35 | 2.24 | 10681 |
| 16 | -1.82 | -2.04 | -1.52 | 1.07 | 1.10 | 10500 |
| 17 | -1.84 | -2.20 | -1.23 | 1.16 | 1.49 | 10588 |
| 18 | -2.16 | -2.45 | -1.89 | 1.38 | 2.36 | 10500 |
| 19 | -1.49 | -2.11 | -0.75 | 1.16 | 1.48 | 10220 |
| 20 | -1.73 | -2.08 | -1.35 | 1.17 | 1.28 | 10500 |
| 21 | -2.05 | -2.58 | -1.66 | 1.43 | 2.47 | 10500 |
| 22 | -2.34 | -3.08 | -1.33 | 1.14 | 1.18 | 10500 |
| 23 | -2.14 | -2.32 | -2.00 | 1.88 | 3.68 | 10500 |
| 24 | -1.96 | -2.01 | -1.85 | 1.35 | 2.25 | 10006 |
| 25 | -1.77 | -2.13 | -1.33 | 1.35 | 1.95 | 10816 |
| 26 | -1.92 | -2.52 | -0.99 | 1.31 | 1.99 | 10500 |
| 27 | -2.12 | -2.27 | -1.98 | 1.11 | 1.34 | 10208 |
|  |  |  |  |  |  |  |
| ***Difusion in forest*** $\boldsymbol{(}\boldsymbol{D}_{\boldsymbol{i}}^{\boldsymbol{1}}\boldsymbol{)}$ | | | |  |  |  |
| **Individual** $\boldsymbol{i}$ | **Mean** | **p 0.025** | **p 0.975** | **Rhat** | **95% C.I.** | **n.eff** |
| 1 | 11.99 | 11.02 | 12.57 | 1.12 | 1.37 | 10500 |
| 2 | 12.78 | 12.59 | 12.87 | 1.32 | 2.16 | 10500 |
| 3 | 12.99 | 12.94 | 13.03 | 1.43 | 3.84 | 10500 |
| 4 | 12.52 | 11.66 | 13.00 | 1.10 | 1.28 | 10721 |
| 5 | 12.58 | 11.88 | 12.99 | 1.06 | 1.14 | 10500 |
| 6 | 12.91 | 12.53 | 13.17 | 1.18 | 1.63 | 10815 |
| 7 | 12.65 | 11.59 | 13.24 | 1.10 | 1.24 | 10500 |
| 8 | 12.54 | 11.57 | 13.00 | 1.07 | 1.10 | 10368 |
| 9 | 12.55 | 12.03 | 13.00 | 1.38 | 2.39 | 11226 |
| 10 | 12.67 | 12.49 | 13.00 | 1.10 | 1.17 | 10416 |
| 11 | 12.88 | 12.38 | 13.07 | 1.43 | 2.41 | 10423 |
| 12 | 12.74 | 12.40 | 13.24 | 1.08 | 1.16 | 10986 |
| 13 | 12.68 | 12.44 | 12.99 | 1.39 | 2.37 | 10500 |
| 14 | 12.52 | 11.96 | 12.96 | 1.58 | 2.92 | 10500 |
| 15 | 12.18 | 11.27 | 13.51 | 1.45 | 3.38 | 10865 |
| 16 | 12.68 | 11.72 | 13.18 | 1.18 | 1.46 | 10583 |
| 17 | 12.58 | 11.28 | 13.87 | 1.21 | 1.78 | 10500 |
| 18 | 12.43 | 11.73 | 13.25 | 1.41 | 2.53 | 10323 |
| 19 | 12.32 | 11.91 | 13.02 | 2.27 | 4.12 | 10500 |
| 20 | 12.66 | 11.16 | 13.96 | 1.21 | 1.60 | 10500 |
| 21 | 12.80 | 12.49 | 13.12 | 1.28 | 1.83 | 11516 |
| 22 | 12.66 | 11.51 | 13.39 | 1.08 | 1.27 | 10500 |
| 23 | 12.38 | 11.62 | 13.00 | 1.02 | 1.06 | 10500 |
| 24 | 12.93 | 12.59 | 13.05 | 1.49 | 3.85 | 12116 |
| 25 | 12.60 | 12.41 | 12.97 | 1.63 | 3.93 | 10624 |
| 26 | 13.27 | 13.00 | 13.87 | 1.18 | 1.64 | 10500 |
| 27 | 12.75 | 12.29 | 13.18 | 1.29 | 1.82 | 10500 |
|  |  |  |  |  |  |  |
| ***Difusion in matrix*** $\boldsymbol{(}\boldsymbol{D}_{\boldsymbol{i}}^{\boldsymbol{2}}\boldsymbol{)}$ | | | |  |  |  |
| **Individual** $\boldsymbol{i}$ | **Mean** | **p 0.025** | **p 0.975** | **Rhat** | **95% C.I.** | **n.eff** |
| 1 | 12.85 | 12.33 | 13.43 | 2.20 | 5.21 | 10229 |
| 2 | 12.71 | 12.14 | 13.06 | 1.13 | 1.36 | 10885 |
| 3 | 12.79 | 12.32 | 13.00 | 1.25 | 1.72 | 10500 |
| 4 | 12.65 | 12.00 | 13.00 | 1.34 | 2.20 | 10500 |
| 5 | 12.29 | 11.43 | 13.00 | 1.33 | 2.15 | 11425 |
| 6 | 13.12 | 12.62 | 13.61 | 2.30 | 4.86 | 10707 |
| 7 | 12.89 | 12.55 | 13.22 | 1.59 | 3.36 | 10500 |
| 8 | 13.03 | 12.44 | 13.62 | 1.50 | 2.47 | 10655 |
| 9 | 12.82 | 12.19 | 13.18 | 1.12 | 1.14 | 10158 |
| 10 | 12.82 | 11.54 | 13.98 | 1.16 | 1.30 | 10648 |
| 11 | 12.73 | 11.90 | 13.86 | 1.24 | 1.75 | 11639 |
| 12 | 12.66 | 11.35 | 13.04 | 1.08 | 1.21 | 10189 |
| 13 | 12.57 | 12.23 | 13.02 | 1.73 | 2.88 | 10500 |
| 14 | 12.69 | 12.48 | 12.99 | 1.30 | 1.99 | 11095 |
| 15 | 12.58 | 11.82 | 12.92 | 1.30 | 1.86 | 10500 |
| 16 | 12.55 | 11.88 | 13.02 | 1.12 | 1.35 | 10818 |
| 17 | 13.15 | 12.33 | 13.91 | 1.17 | 1.53 | 10896 |
| 18 | 12.36 | 12.00 | 12.95 | 1.45 | 2.31 | 10323 |
| 19 | 12.67 | 11.72 | 13.35 | 1.11 | 1.32 | 11012 |
| 20 | 12.80 | 12.50 | 13.07 | 1.10 | 1.29 | 10500 |
| 21 | 13.09 | 12.41 | 14.18 | 1.37 | 2.03 | 10500 |
| 22 | 12.65 | 12.34 | 13.00 | 1.26 | 2.04 | 10075 |
| 23 | 12.57 | 11.82 | 13.08 | 1.27 | 1.87 | 10617 |
| 24 | 12.86 | 12.56 | 13.04 | 1.29 | 2.01 | 10500 |
| 25 | 12.22 | 11.44 | 13.01 | 1.49 | 2.33 | 10276 |
| 26 | 12.85 | 12.46 | 13.02 | 1.28 | 1.76 | 11007 |
| 27 | 12.42 | 11.83 | 12.94 | 1.34 | 2.85 | 11263 |
|  |  |  |  |  |  |  |
| ***Difusion in open areas*** $\boldsymbol{(}\boldsymbol{D}_{\boldsymbol{i}}^{\boldsymbol{3}}\boldsymbol{)}$ | | |  |  |  |  |
| **Individual** $\boldsymbol{i}$ | **Mean** | **p 0.025** | **p 0.975** | **Rhat** | **95% C.I.** | **n.eff** |
| 1 | 12.44 | 11.64 | 13.39 | 1.33 | 2.31 | 10099 |
| 2 | 12.37 | 10.71 | 13.01 | 1.26 | 1.98 | 10317 |
| 3 | 12.16 | 11.30 | 12.94 | 1.20 | 1.58 | 12177 |
| 4 | 12.24 | 11.81 | 13.02 | 1.73 | 4.22 | 10278 |
| 5 | 12.62 | 12.34 | 13.00 | 1.24 | 1.81 | 10500 |
| 6 | 12.13 | 10.78 | 12.90 | 1.19 | 1.59 | 10237 |
| 7 | 12.39 | 12.09 | 12.75 | 1.07 | 1.10 | 10692 |
| 8 | 11.74 | 10.47 | 13.08 | 1.49 | 3.03 | 10694 |
| 9 | 11.91 | 11.18 | 12.99 | 1.25 | 1.81 | 10500 |
| 10 | 12.46 | 11.95 | 12.98 | 1.10 | 1.15 | 10482 |
| 11 | 12.26 | 11.32 | 13.00 | 1.12 | 1.38 | 10500 |
| 12 | 12.48 | 11.69 | 12.99 | 1.16 | 1.47 | 10816 |
| 13 | 12.47 | 11.33 | 12.99 | 1.11 | 1.22 | 10850 |
| 14 | 12.39 | 11.61 | 13.36 | 1.24 | 1.96 | 10938 |
| 15 | 12.05 | 11.46 | 13.07 | 1.32 | 1.99 | 10500 |
| 16 | 12.49 | 12.06 | 13.00 | 1.18 | 1.58 | 10500 |
| 17 | 12.51 | 11.95 | 13.02 | 1.39 | 2.00 | 10500 |
| 18 | 12.01 | 10.28 | 12.99 | 1.16 | 1.48 | 10500 |
| 19 | 13.01 | 12.80 | 13.38 | 1.20 | 1.72 | 10500 |
| 20 | 12.78 | 11.69 | 13.63 | 1.86 | 3.85 | 10322 |
| 21 | 12.40 | 11.78 | 12.99 | 1.38 | 2.11 | 10500 |
| 22 | 12.33 | 10.86 | 13.01 | 1.08 | 1.24 | 10290 |
| 23 | 12.62 | 11.62 | 13.00 | 1.09 | 1.29 | 10661 |
| 24 | 12.45 | 11.22 | 13.04 | 1.07 | 1.10 | 10512 |
| 25 | 12.76 | 12.39 | 13.03 | 1.33 | 2.06 | 10500 |
| 26 | 12.13 | 10.40 | 13.01 | 1.34 | 2.35 | 10500 |
| 27 | 12.71 | 11.73 | 13.20 | 1.05 | 1.13 | 10946 |
|  |  |  |  |  |  |  |
| ***Detection probability*** $\left( \boldsymbol{q}_{\boldsymbol{i}} \right)$ | | |  |  |  |  |
| **Individual** $\boldsymbol{i}$ | **Mean** | **p 0.025** | **p 0.975** | **Rhat** | **95% C.I.** | **n.eff** |
| 1 | 1.55 | -0.23 | 2.32 | 1.29 | 2.32 | 10324 |
| 2 | 2.07 | 1.71 | 2.32 | 1.79 | 3.55 | 10322 |
| 3 | 1.75 | 1.43 | 2.19 | 1.20 | 1.61 | 10500 |
| 4 | 2.33 | 2.08 | 2.62 | 1.21 | 1.71 | 10734 |
| 5 | 1.63 | 0.90 | 2.26 | 1.29 | 2.01 | 10434 |
| 6 | 2.23 | 1.87 | 2.39 | 1.27 | 1.86 | 10500 |
| 7 | 1.55 | 1.19 | 2.19 | 1.05 | 1.12 | 10126 |
| 8 | 2.00 | 1.00 | 2.73 | 1.20 | 1.60 | 10327 |
| 9 | 1.79 | 1.36 | 2.37 | 1.21 | 1.79 | 10670 |
| 10 | 1.09 | 0.60 | 2.01 | 1.44 | 2.26 | 10762 |
| 11 | 2.10 | 1.61 | 2.42 | 1.09 | 1.16 | 10500 |
| 12 | 1.76 | 1.32 | 2.36 | 1.28 | 1.84 | 10500 |
| 13 | 2.11 | 1.97 | 2.20 | 1.22 | 1.78 | 11107 |
| 14 | 2.46 | 2.16 | 2.98 | 2.10 | 4.07 | 10170 |
| 15 | 2.47 | 1.64 | 3.66 | 1.63 | 3.07 | 10030 |
| 16 | 2.19 | 1.46 | 3.32 | 1.10 | 1.33 | 10500 |
| 17 | 2.33 | 2.16 | 2.69 | 1.92 | 4.34 | 10500 |
| 18 | 2.07 | 1.85 | 2.20 | 1.91 | 4.59 | 10500 |
| 19 | 2.04 | 0.76 | 2.80 | 1.22 | 1.60 | 10820 |
| 20 | 1.83 | 0.52 | 2.52 | 1.15 | 1.45 | 10322 |
| 21 | 2.12 | 1.16 | 2.46 | 1.18 | 1.57 | 11062 |
| 22 | 1.65 | 0.80 | 2.56 | 1.48 | 4.21 | 10500 |
| 23 | 1.61 | 0.92 | 2.26 | 1.55 | 3.21 | 10500 |
| 24 | 2.21 | 2.03 | 2.46 | 1.25 | 1.88 | 10728 |
| 25 | 1.54 | 0.82 | 2.73 | 1.07 | 1.16 | 10922 |
| 26 | 3.06 | 1.49 | 5.15 | 1.60 | 4.55 | 9749 |
| 27 | 1.72 | 0.98 | 2.20 | 1.42 | 2.17 | 10500 |
